# StCDF1: A ‘*jack of all trades*’ clock output with a central role in regulating potato nitrate reduction activity

**DOI:** 10.1111/nph.20186

**Published:** 2024-11-06

**Authors:** Maroof Ahmed Shaikh, Lorena Ramírez‐Gonzales, José M. Franco‐Zorrilla, Evyatar Steiner, Marian Oortwijn, Christian W. B. Bachem, Salomé Prat

**Affiliations:** ^1^ Centre for Research in Agricultural Genomics (CRAG) Barcelona 08193 Spain; ^2^ Plant Breeding Wageningen University & Research PO Box 386 Wageningen AJ 6700 the Netherlands; ^3^ Departamento de Genética Molecular de Plantas Centro Nacional de Biotecnología – CSIC Madrid 28049 Spain; ^4^ Solynta Dreijenlaan 2 Wageningen HA 6703 the Netherlands

**Keywords:** DAP‐seq, nitrate reductase, promoter polymorphism, *Solanum tuberosum* L., *StCDF1* transcription factor

## Abstract

- Transcription factors of the CYCLING DOF FACTOR (CDF) family activate in potato the SP6A FT tuberization signal in leaves. In modern cultivars, truncated StCDF1.2 alleles override strict SD control by stabilizing the StCDF1 protein, which leads to StCOL1 suppression and impaired activation of the antagonic SP5G paralog.
- By using DAP‐seq and RNA‐seq studies, we here show that StCDF1 not only acts as an upstream regulator of the day length pathway but also directly regulates several N assimilation and transport genes.
- StCDF1 directly represses expression of NITRATE REDUCTASE (NR/NIA), which catalyses the first reduction step in nitrate assimilation, and is encoded by a single potato locus. *StCDF1* knock‐down lines performed better in N‐limiting conditions, and this phenotype correlated with derepressed *StNR* expression. Also, deletion of the StNR DAP‐seq region abolished repression by StCDF1, while it did not affect NLP7‐dependent activation of the *StNR* promoter.
- We identified multiple nucleotide polymorphisms in the DAP‐seq region in potato cultivars with early *StCDF1* alleles, suggesting that this genetic variation was selected as compensatory mechanism to the negative impact of StCDF1 stabilization. Thereby, directed modification of the StCDF1‐recognition elements emerges as a promising strategy to enhance limiting StNR activity in potato.

## Introduction

All life on earth is exposed to dramatic changes in the light over the 24‐h day : night cycle that vary with the progression of annual seasons. Living organisms have accordingly evolved internal diel systems to synchronize their growth and metabolism to these oscillating conditions. Plants use light as an energy source and thus are even more dependent on this external cue than animals. Both, carbon assimilation and uptake of water and nutrients from soil are diurnally regulated, loss of this circadian rhythmicity being shown to be associated with reduced plant biomass and viability (Kim *et al*., 2017; Flis *et al*., 2019).

Tuber formation is an adaptive strategy of potato plants for winter survival. Shorter day lengths and cooler temperatures are perceived by the plant as indicative of approaching winter, hence promoting formation of these belowground modified shoots (Hannapel *et al*., 2017). Formation of these organs depends on a day length cascade closely related to the *Arabidopsis* flowering pathway, but that in potato activates a negative *FLOWERING LOCUS T* (FT)‐like signal (Abelenda *et al*., 2016). The clock output CYCLING DOF FACTOR1 (StCDF1) has a central role in this pathway by directly repressing the potato *CONSTANS‐like* (*StCOL1*) gene (Kloosterman *et al*., 2013). In long days (LDs), *StCOL1* is expressed in the day, and the protein is phyB‐stabilized, hence activating *SP5G*, a negative FT‐like gene suppressing activation of the FT SP6A tuberization signal in the leaves (Abelenda *et al*., 2016). In short days (SDs), *StCOL1* expression coincides with late night and rapid dark destabilization of the protein prevents *SP5G* activation, leading to *SP6A* derepression via a yet poorly understood mechanism (Abelenda *et al*., 2016). StCDF1 is moreover destabilized on interaction with GIGANTEA (GI) and FLAVIN‐BINDING KELCH REPEAT F‐BOX (FKF1), which in *Arabidopsis* leads to elevated *AtCO* expression during LD afternoon (Sawa *et al*., 2007). This post‐transcriptional control is responsible in potato for the strict SD tuberization control of wild species, while this control is evaded by the *earliness* locus encoding truncated StCDF1 variants (*StCDF1.2* and *StCDF1.3*) that lack the C‐terminal FKF1‐interacting domain (Kloosterman *et al*., 2013). These alleles lead to StCDF1 protein accumulation and sustained *StCOL*s repression, thereby allowing for *SP6A* upregulation in LDs, while they were strongly selected after the introduction of potato into Europe (Hardigan *et al*., 2017; Gutaker *et al*., 2019). Early alleles are widely spread in modern ware and processing germplasm, while late starch potatoes carry the wild‐type (WT) isoform (Hoopes *et al*., 2022).

Studies in *Arabidopsis* established a role of AtCDF1 in nitrogen‐regulated gene expression, with this factor found to share many downstream targets with the N‐signalling NIN‐LIKE PROTEIN 7 (AtNLP7) master regulator (Marchive *et al*., 2013; Varala *et al*., 2018). Tomato rootstocks overexpressing the *Arabidopsis* AtCDF3 gene were also observed to improve yield under low N inputs of grafted WT tomato scions, although a similar effect was not observed for *SlCDF3* possibly due to reduced mobility of this transcript (Begoña *et al*., 2024). An elevated N‐fertilizer supply is on the other hand required in potato for high tuber yields, because low nitrogen use potato efficiency and shallow roots (Tiwari *et al*., 2018). However, when applied too late, nitrate inhibits tuberization, suggesting that N‐signalling negatively affects the day length pathway via a not yet understood molecular mechanism (Li *et al*., 2016).

Nitrogen (N) is an essential plant nutrient, as it is a key component of proteins, Chl, nucleic acids, and many secondary metabolites. Nitrate is the main source of nitrogen for most plants, while its use implies a multistep process including nitrate uptake by roots, its distribution to the rest of the plant, and further assimilation within the cell, in addition to its later remobilization upon leaf senescence (Wang *et al*., 2018). Nitrate uptake and distribution is coordinated by members of the NITRATE TRANSPORTER (*NRT*) family, among which the *NRT1* family members comprise a low‐affinity transport system (LATS) acting under high nitrate. An exception is *NRT1.1* (*CHL1*) that on phosphorylation changes its activity to a high‐affinity transporter and acts as a nitrate sensor, activating the master regulator NIN‐LIKE PROTEIN 7 (NLP7) (Marchive *et al*., 2013; Liu *et al*., 2017). The NLP family proteins also modulate the expression of genes encoding other TFs, such as the *NITRATE‐INDUCIBLE GARP‐TYPE TRANSCRIPTIONAL REPRESSOR 1* (*NIGT1*) family, which further represses *NRT2.1*, *NRT2.4*, and nitrate uptake (Konishi & Yanagisawa, 2014; Ueda *et al*., 2020). Another family of nitrate‐regulated TFs, *LATERAL ORGAN BOUNDARY* (*LBD*), is additionally shown to modulate nitrate uptake and transport by transcriptionally inhibiting *OsNRT2.1*, *OsNRT2.2*, and *OsNRT2.3* in rice (Zhu *et al*., 2022; Jiang *et al*., 2023). Many NRT1s transport not only nitrate but also glucosinolates and hormones (Kanno *et al*., 2012; Nour‐Eldin *et al*., 2012), being shown to play a relevant role in plant abiotic stress adaptation (Ye *et al*., 2022). The *NRT2* family members in turn comprise a high‐affinity transport system (HATS) involved in nitrate acquisition under low‐nitrate concentrations, hence playing an essential role in N deprivation conditions (Ruffel *et al*., 2021). Once inside the cell, nitrate is initially reduced to nitrite by cytosolic NITRATE REDUCTASE (NR), which catalyses a rate‐limiting step in nitrate assimilation (Liu *et al*., 2022). Subsequently, nitrite is translocated into the chloroplast, being reduced by NITRITE REDUCTASE (NiR) into ammonium. This is toxic to plant cells and rapidly incorporated into carbon skeletons through the combined action of glutamine synthetase (GS) and glutamate synthase (GOGAT), forming the GS/GOGAT cycle (Glass, 2003; Masclaux‐Daubresse *et al*., 2010). Glutamate dehydrogenase (GDH) and l‐asparaginase contribute also to the conjugation of ammonia to amino acids, at the expense of shoot‐derived carbohydrates (Bernard & Habash, 2009). A complex regulatory network regulates transcription of these genes and coordinates root and shoot development in response to N availability (Gaudinier *et al*., 2018; Varala *et al*., 2018; Brooks *et al*., 2019). Different N deprivation‐responsive genes were also identified in potato leaves, stolons, or roots, underscoring that response to low N varies between tissues (Tiwari *et al*., 2020; Zhang *et al*., 2020).

Here, we analysed the molecular targets of StCDF1 by using DNA‐binding affinity purification followed by high‐throughput sequencing (DAP‐seq) (Franco‐Zorrilla & Prat, 2021). Combination of this binding data set with RNA‐seq transcriptomic data from potato *StCDF1oe* and RNAi lines revealed that this factor modulates a broad range of biological processes including nitrogen‐related gene expression. We show that *StCDF1* represses the single potato *NITRATE REDUCTASE* (*NR/NIA1*) gene, with StCDF1 down‐regulation improving plant performance in low N.

## Materials and Methods

### Plant material

The *Solanum tuberosum* L. diploid genotype CE3027, an offspring of the C (USW5337.3) × E (VPH4 77.2102.37) population, was used as the WT background, homozygous for *StCDF1.1*. For RNA‐seq studies, previously described *StCDF1_RNAi* #13 and *35S:StCDF1.2 #10* lines (10) were used, along with the nontransformed CE3027 WT controls.

Plants were initially propagated in tissue culture and transplanted into soil, to be further grown in climate chambers under SDs. After 4 wk, the third leaf from the apex was sampled at ZT1. In total, two experimental replicates were used to these analyses, each replicate comprising a pool of three independent plants (biological replicates). Leaf samples were frozen in liquid nitrogen and stored at −80°C until RNA extraction. For low N *in vitro* growth studies, lines overexpressing *StCDF1.1* (*35S:StCDF1.1*#25, *35S:StCDF1.1*#30) and knock‐down *StCDF1* lines (*StCDF1 RNAi* #83, *StCDF1 RNAi* #88) were generated according to Kloosterman *et al*. (2013), and grown together with nontransformed (CE3027) controls.

### RNA‐seq studies

Total RNA was extracted with the RNeasy Plus Mini kit (Qiagen). RNA was quantified using a NanoDrop 1000 v.3.7 (Thermo Fisher Scientific, Wilmington, DE, USA), and its quality was evaluated by agarose gel electrophoresis before sequencing. RNA sequencing was performed at Novogene Bioinformatics Technology Co., Ltd (Beijing, China) on an Illumina HiSeq 2500 system (Illumina Inc., San Diego, CA, USA), to the generation of 2 × 150 bp pair‐ended reads, and a sequencing coverage of at least 6 GB per replicate. Raw data quality was evaluated by using the fastqc software, and reads for each experimental replicate were mapped into the DM 1‐3516 R44 (Sol.Tub_3.0) reference genome using HISAT2 (Kim *et al*., 2015). Counts per transcript (Fragments Per Kilobase of transcript per Million mapped reads (FPKM)) were obtained by HTSeq‐count (Anders *et al*., 2015), and analysis of differential gene expression between *StCDF1oe*, *StCDF1‐RNAi*, and the nontransformed controls was conducted with DESeq2. A log_2_FC > 1 and *P* < 0.05 was used as cut‐off threshold for differential expression.

### DAP‐seq Illumina library preparation and DNA‐binding affinity purification

DNA Affinity Purification and sequencing (DAP‐seq) studies were carried out as in Bartlett *et al*. (2017), with some modifications (Franco‐Zorrilla & Prat, 2021). Genomic DNA was extracted from stolons of *S. tuberosum group andigena* (genotype 7540) plants using the CTAB method, digested with RNAseA, and subsequently used for preparation of the DAP‐seq libraries. The gDNA was fragmented in a Covaris M220 ultrasonicator to 200 bp and subjected to DNA‐ends correction and A‐tail creation, before Y adapter ligation. Y adapters were generated by annealing Strand A: 5′‐ACACTCTTTCCCTACACGACGCTCTTCCGATCT‐3′ and Strand B: 5′‐(P)‐GATCGGAAGAGCACACGTCTGAACTCCAGTCAC‐3′ primers, before the ligase reaction. Both *E. coli* expressed full‐length StCDF1.1 (Experiments 1 and 2) and truncated StCDF1.2 (Experiment 3) proteins were used for binding of the gDNA libraries. To this purpose, the *StCDF1.1* and *StCDF1.2* coding regions were C‐terminally fused to the maltose‐binding protein (MBP) in the pDEST‐TH1 vector. *E. coli* BL21 cells were then transformed with these constructs, and cultures were induced with 1 mM Isopropyl β‐d‐1‐thiogalactopyranoside (IPTG) to be subsequently overnight incubated at 16°C for expression of the respective recombinant proteins. 25 ml aliquots of these overnight cultures were pelleted and kept frozen at −80°C until use. Soluble proteins were extracted from these cells by lysis in 1× PBS buffer, and 400 μl of cleared extracts were incubated with 400 ng of the potato gDNA library, prior purification of the protein–DNA complexes on Amylose Magnetic Beads (New England Biolabs, Ipswich, MA, USA), following the manufacturer's instructions. DNA recovered from the beads was then PCR‐amplified for 20 cycles with primer A (Illumina TruSeq Universal_Primer): 5′‐AATGATACGGCGACCACCGAGATCTACACTCTTTCCCTACACGACGCTCTTCCGATCT‐3′ and primer B (Illumina TruSeq Index Primer): 5′‐CAAGCAGAAGACGGCATACGAGAT‐NNNNNN‐GTGACTGGAGTTCAGACGTGTGCTCTTCCG‐3′, where the NNNNNN 6‐mer sequence was ‘ATCACG’ for Experiment 1, ‘CGATGT’ for Experiment 2, and ‘ATCACG’ for Experiment 3, respectively. As a negative control, input DNA samples were obtained by directly amplifying the gDNA library for 10 cycles, and cleaning and quantification of the amplification product as the protein bound DNAs. Indexed libraries were sequenced by Novogene (Cambridge Lab, Cambridge, UK), using Illumina HiSeqX (PE 2×150, Experiment 1 and input sample) and MiSeq V3 (PE 2×75, Experiments 2 and 3).

Raw reads in FASTQ format were trimmed to remove the adaptor sequences, and low‐quality reads were filtered out (Phred < 20 and length < 20) with Trim Galore. Paired reads were mapped to the potato PGSC v.4.03 reference genome (https://solgenomics.net/) with bowtie2 (Langmead & Salzberg, 2012), using default parameters. BAM files were then converted to binary BIGWIG interval files with the ‘BAM coverage’ tool in deeptools2 (Ramírez *et al*., 2016), using a bin size of 10 bases, and normalized to bins per million (BPM, equivalent to TPM in RNA‐seq) in the European Galaxy Project portal (https://usegalaxy.eu/). Peak calling was performed with GEM (Guo *et al*., 2022), using the corresponding ‘input’ sample as a negative control, and parameters: ‘‐‐range 200 ‐‐smooth 0 ‐‐mrc 1 ‐‐fold 2 ‐‐q 1.3010 ‐‐k_min 6 ‐‐k_max 20 ‐‐k_seqs 600 ‐‐k_neg_dinu_shuffle ‐‐pp_nmotifs 1’, with BEDTOOLS getfasta (Quinlan, 2014) being used to obtain the 200‐bp sequences from the enriched peaks. The top‐ranking 600 sequences were finally utilized for *de novo* discovery of recognition motifs with MEME, using the ‘‐dna ‐mod zoops ‐nmotifs 1 ‐bfile ‐revcomp ‐minw 8 ‐maxw 20’ command and a Markov model 0 background, calculated from the corresponding genomes.

### StCDF1 target identification

To identify the genes directly regulated by StCDF1, we intersected the RNA‐seq DEG data sets from StCDF1 lines with the generated DAP‐seq data. To this end, we first retrieved all potential StCDF1 targets by selecting each of the genes showing a DAP‐seq peak located within the upstream 5.0 kb region to the translation start site (TSS), in at least two of the three DNA‐binding studies. Genes present in both data sets were selected to define a putative list of direct StCDF1 targets, which was then employed for Gene Ontology (GO) enrichment analyses using the topgo package (http://www.geneontology.org/). InterPro protein domain and KEGG pathway enrichments were performed using shinygo v.0.61. GO terms were considered as significant when their Benjamini–Hochberg false discovery rate (FDR) adjusted *P*‐value was < 0.05. To the generation of the nitrogen‐related target subset, genes that grouped in the GO terms nitrogen metabolism or nitrogen response were complemented with the previously described *Arabidopsis* AtCDF1 targets involved in N homeostasis (Varala *et al*., 2018; Alvarez *et al*., 2020) and those that in *Arabidopsis* or potato RNA‐seq studies were found to be differentially expressed in response to low N (Gaudinier *et al*., 2018; Brooks *et al*., 2019; Tiwari *et al*., 2020; Zhang *et al*., 2020). We designated this final list StCDF1 target nitrogen‐related genes (TargN_2_‐RG).

### Transcript amplification and time course studies

Reverse transcription quantitative polymerase chain reaction studies were used to corroborate that StCDF1 targets were differentially expressed in the *StCDF1* lines. For time course studies, the third leaf from the top was sampled from *StCDF1* knock‐down lines, lines overexpressing *StCDF1*.2 and nontransformed plants. Two experimental replicates were sampled to these studies, each one comprising a pool of three biological replicates. Samples were frozen in liquid nitrogen and stored at −80°C until RNA preparation. RNA was extracted using the RNeasy Plus Mini Kit (Qiagen), according to the suppliers' recommendations and then treated with DNAse using the RNAse‐Free DNAse set from Qiagen. RNAs were measured with a Nanodrop‐ND‐1000 spectrophotometer, and tested for quality by gel electrophoresis, before reverse transcription quantitative polymerase chain reaction. The Transcriptor First strand cDNA synthesis kit (Roche) was used for reverse transcription, 1 μg RNA using primer hexamers, and 6.0 μl the 1/10 diluted reaction used for PCR amplification. Specific primers were designed with the Primer3Plus online software (www.bioinformatics.nl/cgi‐bin/primer3plus/primer3plus.cgi) and the *Elf* (elongation factor) gene used as housekeeper (Supporting Information Dataset [Link], [Link], [Link], [Link], [Link], [Link], [Link]). The relative quantitative method (2^−▵▵CT^) was employed to calculate expression levels of the target genes.

### Growth of *in vitro* *StCDF1* lines in low nitrogen

For low‐nitrogen treatment, we used *in vitro* propagated *StCDF1‐RNAi*, *StCDF1.1oe*, and WT CE3027 plants. Plants were grown at 22°C on Murashige and Skoog (MS)‐modified basal salt media (M531; Phytotechnology Laboratories, Lenexa, KS, USA) containing 1% (w/v) sucrose and 0.8% (w/v) plant agar, under LD (16 h : 8 h, light : dark) photoperiodic conditions. As optimal nitrogen levels (control), we used the MS media with a total 60 mM nitrogen content (Ramage & Williams, 2002). For low N, we used 0.15 mM nitrogen (0.05 mM NH_4_NO_3_ and 0.05 mM KNO_3_), taking as a reference previous work from Yanagisawa *et al*. (2004). Plantlets were weighed for total fresh biomass (g), and the image j software was used for root length (cm) determination after the treatment. Six seedlings per genotype and conditions were used in these experiments. After 10 d of low‐nitrogen exposure, seedlings were sampled for real‐time qPCR analysis.

### Chlorophyll quantification

To determine the Chl content, 12 mg samples was subjected to extraction using 2 ml of 96% ethanol. The resulting extract was then filtered and centrifuged in sealed tubes at 15 000 ***g*** for 5 min. After centrifugation, 300 μl of the supernatant was collected and measured using a spectrophotometer. Ethanol absorbance was used for blank correction. Chl*a* and Chl*b* content in mg g^−1^ of the extracts were calculated according to Lichtenthaler & Buschmann (2001). For extraction, a pool of three biological replicates per genotype and two experimental replicates were used. The entire experiment was conducted three times. For the absorbance measurements, each sample was added in triplicate to the Elisa plate. In total, Chl content results were the means of 18 measurements per sample.

### Transient expression assays

The putative promoter regions of the nitrate reductase (*StNR*) and the nitrate transporter 1/*/PTR FAMILY 3.1* (*StNPF3.1*) genes were amplified from wild *andigena* potato by using primers described in the Dataset S6. A 3 kb and 2.5 kb region upstream of the translation start site (TSS) was, respectively, used, including the StCDF1 DNA‐binding peak obtained from the DAP‐seq analysis (Figs S6, S7). The PCR amplification products were cloned into the GATEWAY cloning vector pDONR207 and later inserted via the LR clonase reaction (Invitrogen) in front of the luciferase reporter in the pGWB435 destination vector. To avoid background activity originated by basal promoter expression in Agrobacteria, a modified version of this vector (pGWBiLUC), bearing the potato L700 intron inserted into the LUC coding region was used in these studies. These reporter constructs were transformed in *Agrobacterium tumefaciens* strain (AGL21) and co‐infiltrated into *Nicotiana benthamiana* (*rdr6i*) leaves with the *35S:StCDF1.1* (full length) and *35S:StCDF1.2* (truncated) effector constructs. *Agrobacterium* strains containing the *pStNR:iLUC* and *pStNPF3.1:iLUC* fusion, as well as *35S:StCDF1.1* and *35S:StCDF1.2* constructs, were resuspended in 10 mM MES (pH 5.8), 10 mM MgSO4 and 150 μM acetosyringone, and 0.2 OD_600_ for all the Agrobacteria cultures were used for infiltration. Two days post infiltration discs (12 discs per treatment) taken from the infiltrated leaves and incubated in 2MS plant medium supplemented with 0.02 mg ml^−1^ d‐luciferin (Promega). Luciferase activity was recorded every 30 mins in Centro LB‐960 microplate luminometer (Berthold Technologies GmbH & Co. KG, Bad Wildbad, Germany), using the mikrowin 2000 (v.4.29) software.

### Nitrate quantification

Estimation of the nitrate content of leaf samples was carried out using established methods (Cataldo *et al*., 1975). 200 mg of leaf samples grown in *in vitro* conditions and soil was weighed in 1.5‐ml microcentrifuge tubes and 1 ml of Milli‐Q water was added for extraction. Samples were homogenized for 5 min and then centrifuged at 15 000 ***g*** for 10 min at room temperature. For estimation of nitrates, 100 μl of the supernatant was added to 400 μl reagent (5% salicylic acid in conc. H_2_SO_4_) in a deep‐well plate and allowed to stand at room temperature for 20 min. 50 μl of this solution was mixed with 250 μl of 2 N NaOH in a 96‐well plate and measured at 410 nm using a plate reader to determine the concentration of nitrates. Nitrates were quantified using a standard curve method, based on the intensity of the developed yellow colour (modified from Cataldo *et al*., 1975).

### Analysis of nitrate reductase enzyme activity

Leaf tissues (100 mg) were collected from the following potato lines: CE3027, *35S:StCDF1.1* #25 & #30, and *StCDF1‐RNAi* #83 & #88. The samples were homogenized in 500 μl of prechilled 0.1 M potassium phosphate buffer (pH 7.5). The homogenates were centrifuged at 12 000 ***g*** for 10 min at 4°C. 150 μl of this soluble protein extract was mixed with 850 μl of reaction buffer composed of 0.1 M potassium phosphate (pH 7.5), 10 μM KNO₃, and 0.27 μM DPNH (NADH) and incubated for 30 min at room temperature. The reaction was terminated by adding 50 μl of zinc acetate, the entire mixture was then centrifuged at 12 000 ***g*** for 10 min, and the supernatant was used to determine NR activity.

A freshly prepared solution mixture containing 1% (w/v) sulphanilamide in 3 N HCl and 0.05% N‐(1‐naphthyl) ethylenediamine hydrochloride (NED) was used for the assay. The absorbance of the resulting pink colour was measured at 540 nm. The nitrite content was quantified using a standard curve prepared with potassium nitrite (KNO₂) (adapted from Agnihotri & Seth, 2016).

### Statistical analysis

Data analysis was performed using the IBM spss software (v.22; https://www.ibm.com/analytics/spss‐statistics‐software). The data obtained from the phenotypic variables and gene expression were subjected to analysis of variance homogeneity to determine whether its distribution was normal. Since the data presented non‐normal distribution, we applied nonparametric test by using Kruskal–Wallis Test. The differences among the genotypes were found through Fisher's LSD *post hoc* test. The differences were considered significant for a value of *P* ≤ 0.05.

## Results

### Genome‐wide identification of StCDF1 binding sites

**Fig. 1:**
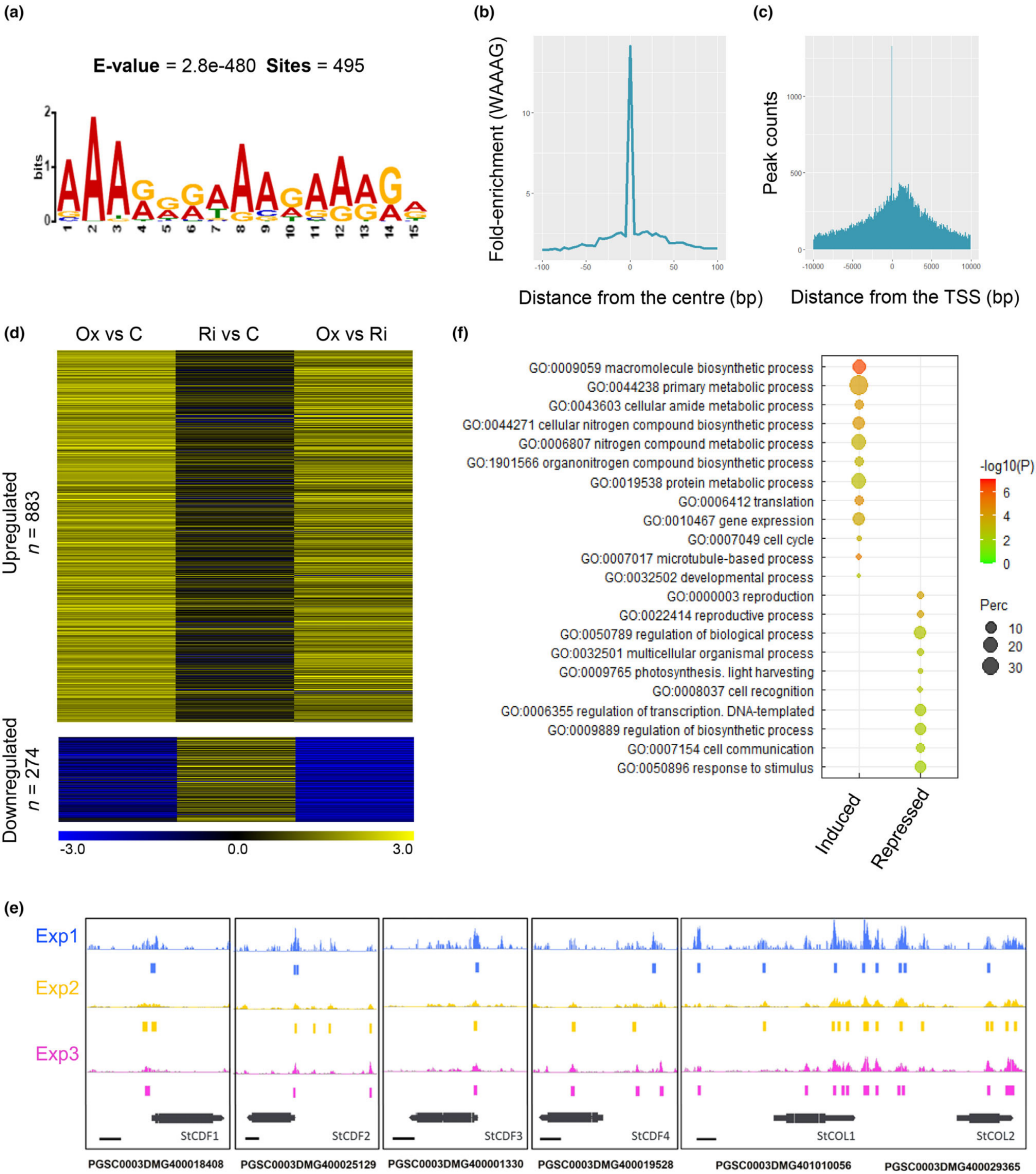
Potato StCDF1 positively and negatively modulates target gene expression. (a) Consensus sequence for StCDF1 binding generated from the 600 more significant peaks. (b) The StCDF1‐binding motif (5′ WAAAG 3′, being W = A or T) is enriched at the centre of the peaks. (c) DAP‐seq peaks are enriched in proximal promoters, near to the translation start site (TSS). (d) Heatmap of directly StCDF1‐activated and StCDF1‐repressed targets as obtained from the combination of RNA‐seq and DAP‐seq results. Target genes were defined as those with at least one StCDF1‐binding peak in their 5 kb upstream region. Ox: Line overexpressing *StCDF1.2*, Ri: *StCDF1* knock‐down; and CT: nontransformed controls (CE3027). (For more details, see Supporting Information Dataset S3). (e) DAP‐Seq StCDF1 binding peaks in the putative promoter regions of the *StCDF1*, *StCDF2*, *StCDF3*, *StCDF4*, *StCOL1*, and *StCOL2* genes. Diagram description from the top: Experiment 1 (blue bar chart), Experiment 2 (yellow bar chart), and Experiment 3 (magenta bar chart). Below the bar charts, significant binding sites are shown as coloured boxes. Gene diagrams are represented at the bottom in black. Bar, 1 kb (left). (f) Summary of the GO term enrichment analyses for the StCDF1 direct targets (full list is provided in Dataset S4).

To identify additional StCDF1 targets to its recognized role in *StCOL*s repression (Kloosterman *et al*., 2013), we analysed the potato genomic regions bound by this factor. To this end, DAP‐seq was carried out on potato genomic DNA, using the bacterial expressed StCDF1 protein. We obtained in three independent experiments 50 632, 92 890, and 117 969 significant peaks, yielding a list of 64 171 higher confidence binding regions present in at least two replicates (Dataset S1). Number of binding loci, though very high, was similar to that obtained for the *Arabidopsis* AtCDF3 protein (8096 peaks), considering the relative sizes of the *Arabidopsis* and potato genomes (Mahajan *et al*., 2012). Analyses of the obtained peaks for *de novo* discovery of recognition elements confirmed the 5′‐YWAAAGRYC‐3′ consensus sequence (Fig. 1a, S1A), earlier identified using a protein‐binding microarray (Ramírez‐Gonzales *et al*., 2021), and showed this motif to be highly enriched in the centre of the peaks (Figs 1b, S1B). Moreover, location of these DAP‐seq peaks with respect to the nearest annotated genes revealed these to be preferentially enriched in proximal promoter regions, close to the TSS (Figs 1c, S1C,D), thus supporting that the *in vitro* experiment reproduced StCDF1 *in vivo* binding activity. By these means, we identified 12 973 putative targets, as defined by the presence of a binding peak within the −5.0 kb to +100 bp region relative to the TSS (Dataset S1, HC peaks _5 kb).

### *StCDF1* acts not only as a canonical repressor, but it activates numerous downstream targets

To investigate relevance of such DNA‐binding events on downstream StCDF1‐regulated gene expression, we performed transcriptomic studies of leaves of plants overexpressing *StCDF1*, RNAi lines, and the untransformed CE3027 WT. Comparison of the RNA‐seq profiles identified 4850 upregulated and 928 downregulated DEGs (log_2_FC > 1) in plants overexpressing the truncated *StCDF1.2* protein with respect to the WT. By contrast, only 1406 genes were up‐ and 316 downregulated in *StCDF1* RNAi vs WT, likely reflecting a mild RNAi silencing. However, 3343 genes were still observed to be up‐ and 1313 downregulated when comparing the *StCDF1*.2*oe* and *StCDF1* RNAi lines (Dataset S2). Surprisingly, upregulated genes were in all these data sets greater than those with a downregulated expression, despite the well‐characterized repressive function of CDF1 (Goralogia *et al*., 2017). This indicated that StCDF1 acts also as a positive regulator of gene expression, or that these genes correspond to indirect targets of this factor.

We thus analysed these putative targets for the presence of StCDF1‐binding peaks in their promoter regions, by intersecting the DAP‐seq and RNA‐seq data sets. This identified 1157 DEGs showing at least one binding peak within the 5.0 kb region upstream the TSS (Dataset S3), which we selected as a reliable set of directly regulated StCDF1 targets. Remarkably, 883 of these genes were upregulated in *StCDF1oe* or downregulated in the *StCDF1*‐RNAi lines, in line with StCDF1 acting as a transcriptional activator. By contrast, only 274 of the directly StCDF1‐bound DEGs were upregulated in *StCDF1*‐RNAi plants (Fig. 1d; Dataset S3), suggesting a repressor activity on fewer targets, although we cannot exclude that more RNAi‐induced genes were identified in stronger silenced lines.

Canonical targets of the day length pathway such as *StCOL*s (Kloosterman *et al*., 2013) and other members of the *StCDF* family were included in this smaller data set (Fig. 1e; Dataset S3), in addition to the homeotic protein *KNOTTED‐1* (*KNOX 1*) and the BEL1‐like homeodomain *BEL14* and *BEL32* factors (Mahajan *et al*., 2012; Sharma *et al*., 2014). These homeodomain genes were up‐ and downregulated, respectively, in *StCDF1oe* lines (Fig. S2; Dataset S3), consistent with StCDF1 directly regulating their expression. These genes were reported to be expressed in sieve elements and their transcripts to be transported to the stolons (Mahajan *et al*., 2012; Sharma *et al*., 2014). Physical KNOX and BEL1 protein interaction facilitates BEL1 nuclear import and the regulation of GA and cytokinin‐related genes by this TF complex, thus reducing GA content while increasing auxin and cytokinin levels in the stolons (Sharma *et al*., 2016).

Additional members of the *StCDF* family, such as *StCDF3* and *StCDF4*, displayed also DAP‐seq peaks in their promoter regions (Fig. 1e) but were not differentially expressed in our RNA profiling analyses. *CDF*s oscillate with characteristic diel patterns, and thus, it is possible that these transcripts peak at a different time point than the one used for RNA‐seq.

Taken together, these findings demonstrate that StCDF1 binds the promoters of a large set of targets and acts not only as a canonical repressor of the day length tuberization pathway, but it activates expression of a broad range of genes involved in biological processes other than tuberization.

### *StCDF1* directly modulates nitrogen‐responsive gene expression

To gain further insight into StCDF1 biological function, DEGs with a binding peak were analysed for their functional annotation. Gene Ontology analyses revealed the upregulated DEGs to be enriched in genes with a role in primary metabolism and biosynthetic processes and related with translation and protein regulation. By contrast, repressed targets were mostly enriched in GO terms related with photosynthetic light harvesting, response to external stimuli and cell–cell communication (Fig. 1f; Dataset S4). The GO term transcriptional regulation was over‐represented in both clusters, these TF targets being likely involved in expanding StCDF1 physiological effects via an indirect control of gene expression. However, the most enriched GO category was nitrogen metabolism and organonitrogen compound biosynthesis, which was significantly over‐represented among the upregulated targets, in line with StCDF1 having a role in modulating potato N homeostasis.

*Arabidopsis* AtCDF1, the closest homolog of *StCDF2*, was identified in transcriptional network analyses as an early N‐responsive TF acting to N‐regulated gene expression downstream of NLP7 (Varala *et al*., 2018; Alvarez *et al*., 2020). Therefore, we retrieved the N‐related genes targeted by StCDF1 by using this transcriptional network, and available low N‐responsive profiles of potato and *Arabidopsis* (Gaudinier *et al*., 2018; Brooks *et al*., 2019; Tiwari *et al*., 2020; Zhang *et al*., 2020). This comparison generated a final list of 160 N‐responsive StCDF1 targets that we designated as target N‐related genes (TargN‐RG; Dataset S5), and which included several nitrate and amino acid transporters (Dataset S5), potato *NITRATE REDUCTASE* (*NR*), and a number of TFs of the WRKY family earlier associated with nutrient starvation (Contento *et al*., 2004; Devaiah *et al*., 2007; Heerah *et al*., 2019). The potato orthologues of *AtWRKY41*, *AtWRKY50*, and *AtWRKY51*, with a role in SA‐responsive gene expression and in antagonizing JA‐signalling (Hussain *et al*., 2018), were here identified as repressed by StCDF1 (Fig. S3). Salicylic acid (SA) has been reported to accumulate in roots in response to low‐N availability (Conesa *et al*., 2020), and it will be interesting in future studies to assess whether these factors functionally link response to low‐N availability and SA signalling.

### *StCDF1* regulates diurnal oscillation of nitrogen‐responsive genes

StCDF1 has a central role in the day length tuberization pathway by regulating cyclic *StCOLs* expression (Kloosterman *et al*., 2013). Notably, *NITRATE REDUCTASE* (*NR*) was reported to be diurnally regulated with other N‐responsive genes (Tucker *et al*., 2004; Gutiérrez *et al*., 2008; Teng *et al*., 2017). We thus investigated whether StCDF1 is involved at modulating cyclic oscillation of these targets, by analysing their diurnal expression in *StCDF1oe* and RNAi lines.

**Fig. 2:**
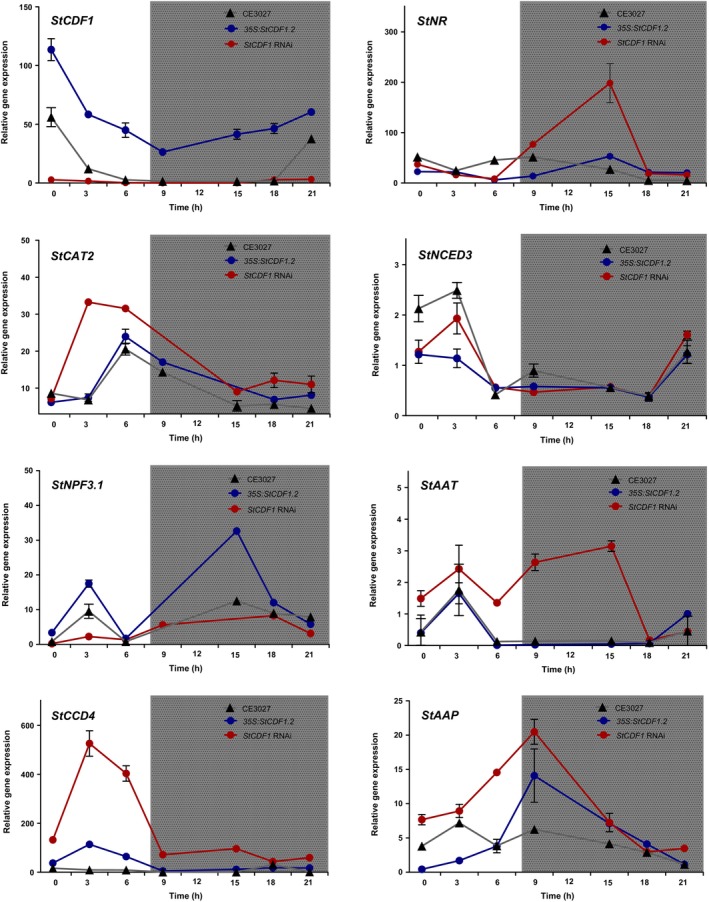
StCDF1 modulates rhythmic expression of nitrogen‐related genes in potato. Time course validation of the target genes up‐ and downregulated by StCDF1 using reverse transcription quantitative polymerase chain reaction. Leaf samples of 4‐wk‐old plants grown in optimal N conditions were used to these studies. Samples were collected every 3 h for a total interval of 24 h. *StCDF1* transcript levels were measured as a control for OE and silencing of this gene. Expression values are the mean ± SE of two biological replicates. *NR: NITRATE REDUCTASE*, *CAT2: CATALASE ISOENZYME 2*, *NPF3.1*: *NITRATE TRANSPORTER1/PTR FAMILY 3.1*, *NCED3*: *9‐CIS‐EPOXYCAROTENOID DIOXYGENASE 3*, *CCD4*: *CAROTENOID CLEAVAGE DIOXYGENASE 4*, *AAT*: *AMINO ACID TRANSPORTER* and *AAP*: *AMINO ACID PERMEASE*.

In a 24‐h time course, *StCDF1* peaked during early morning, in line with previous studies (Kloosterman *et al*., 2013). Peak levels were reduced in RNAi lines, while they were significantly increased in *OE* lines, particularly during late day and early night, coinciding with lower transcription of the endogenous gene (Fig. 2).

Transcript levels of *NITRATE TRANSPORTER 1/PTR FAMILY 3.1* (*StNPF3.1*) were elevated in *StCDF1oe* plants, whereas its morning peak expression was suppressed in RNAi lines, consistently with StCDF1 activating this gene during the day. *NITRATE REDUCTASE* (*NR*) gene expression peaked in CE3027 plants during the day, and in agreement with this gene to be repressed by StCDF1, its peak expression was reduced in overexpression lines. *StNR* transcript levels were in addition strongly upregulated in RNAi lines during early night, indicating that StCDF1 suppresses its expression during night‐time.

Expression of *CAROTENOID CLEAVAGE DIOXYGENASE 4* (*StCCD4*), previously implicated in heat‐induced sprouting and in modulating tuber carotenoid content (Campbell *et al*., 2010), was in addition strongly upregulated in the RNAi lines in the day. *StCCD4* displayed a very prominent DAP‐seq peak, thus validating this gene as one of the strongest StCDF1‐repressed targets during daytime (Fig. 2). We also examined expression of *CATALASE ISOZYME 2* (*StCAT2*), reported in *Arabidopsis* to be regulated by *CDF4* (Xu *et al*., 2020), but filtered out from our final DEGs list by falling below the cut‐off threshold (log_2_FC = −0.995). *StCAT2* showed in RNAi lines a similar upregulated expression in the day as *StCCD4* or *NINE‐CIS‐EPOXYCAROTENOID DIOXYGENASE 3* (*NCED3*), in contrast to other repressed targets as *AMINO ACID TRANSPORTER* (*StAAT*) and *AMINO ACID PERMEASE* (*StAAP*) that were derepressed mostly at night (Fig. 2). Taken together, these results validate our RNA‐seq data (Dataset S2), while they substantiate a role of StCDF1 in the diurnal control of N‐related gene expression.

### A single potato locus encodes *NITRATE REDUCTASE* ( *NR* ) activity

**Fig. 3:**
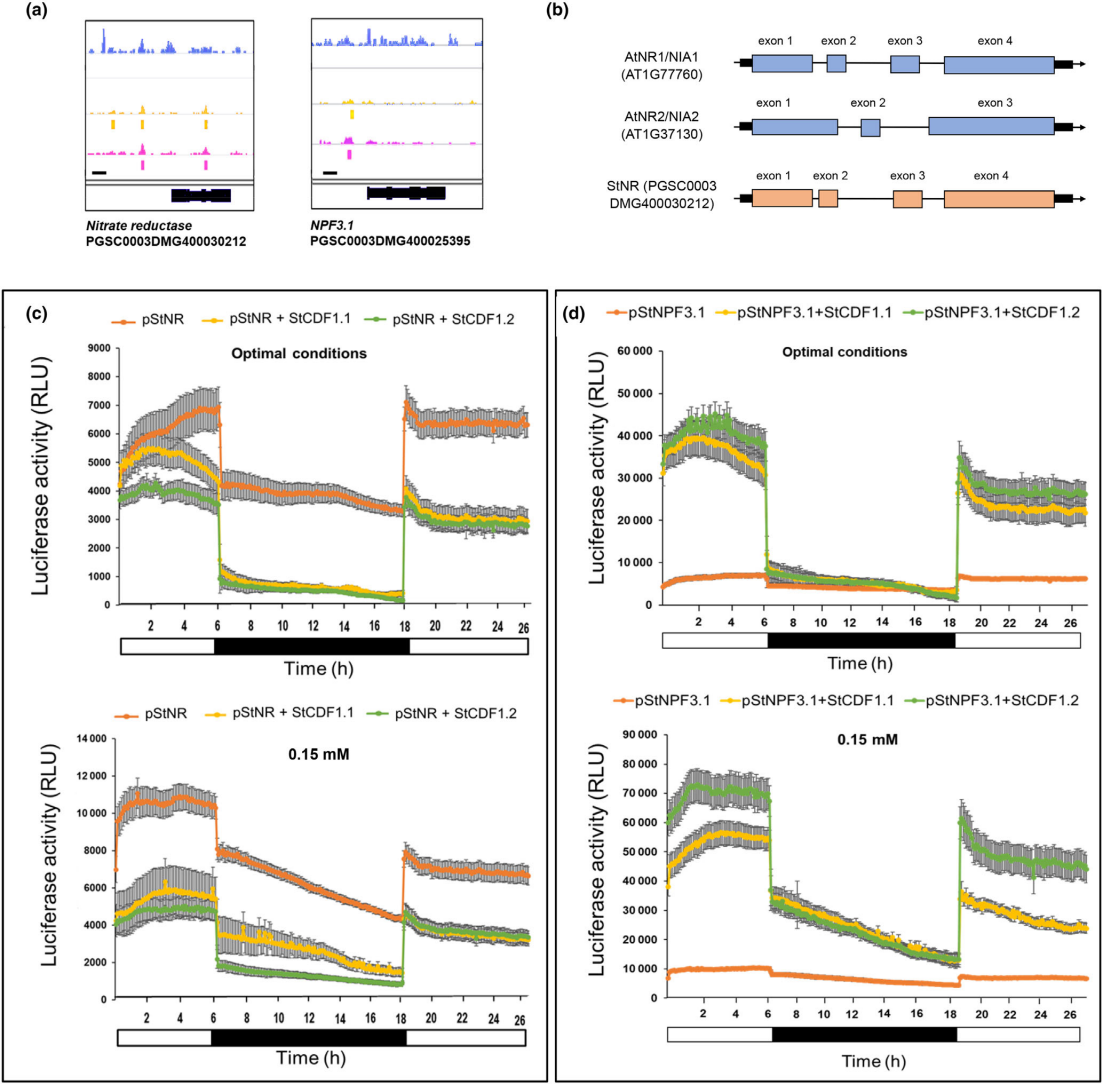
StCDF1 directly controls expression of potato *StNR* and the *StNPF3.1* transporter. (a) DAP‐seq results showing the binding sites of StCDF1 in the putative promoters of *NITRATE REDUCTASE* and *NITRATE TRANSPORTER 1/PTR FAMILY 3.1*. Diagram description from the top: Experiment 1 (blue bar chart), Experiment 2 (yellow bar chart), and Experiment 3 (magenta bar chart). Below the bar charts are represented the significant binding sites obtained from the DAP‐Seq. Gene diagrams are shown in black at the bottom. Bar, 1 kb (left). (b) Intron–exon structure of potato *StNR* and *Arabidopsis AtNIA1* and *AtNIA2* genes. (c, d) Transient transactivation assays in *N. benthamiana* leaves under each optimal and low‐N conditions: (c) Inhibition of *pStNR:iLUC* luciferase activity by *35S:StCDF1.1* and *35S:StCDF1.2* (mean ± SD; *n* = 12). For basal promoter activity, *pStNR:iLUC* was expressed alone. (d) Activation of *pStNPF3.1:iLUC* by *35S:StCDF1.1* and *35S:StCDF1.2* (mean ± SD; *n* = 12). LUC activity of leaves infiltrated with the reporter alone is shown for comparison. For low‐nitrogen conditions, leaf discs were incubated in media containing 0.15 mM nitrate. Data represent the mean ± SD of *n* = 12 discs.

*NITRATE REDUCTASE* (*NR/NIA*) catalyses nitrate reduction, while we observed StCDF1 directly repress this gene (Figs 2, 3a; Dataset S3). StCDF1 also suppressed expression of the *StAAT* and *StAAP* amino acid transporters, while activated *StNPF3.1*, a low‐affinity nitrate transporter implicated in gibberellin transport (David *et al*., 2016; Tal *et al*., 2016). Transcriptional control of these genes hence underscores a role of StCDF1 in negatively regulating N acquisition while promoting nitrate redistribution, aside a possible role in long distance GA transport. GAs have been described to exert an inhibitory effect on tuber formation, and bioactive levels to be reduced in stolons on tuberization transition (Bou‐Torrent *et al*., 2011). Hence, it will be relevant in future studies to assess whether *StNPF3.1* upregulation contributes to GA‐dependent tuberization control.

Reduction in nitrate to nitrite is a limiting step in nitrate assimilation, whereas NR/NIA activity is in most plant species encoded by two or more loci (Berger *et al*., 2020). Potato, tomato, and pepper have, however, a single *NR* locus, in contrast to the two *NtNIA1* and *NtNIA2* tobacco genes (Yang *et al*., 2019, Fig. S4). High‐level identity of the potato StNR, AtNR1/NIA1, and AtNR2/NIA2 proteins prevented us from identifying its *Arabidopsis* ortholog (Fig. S5). Indeed, *StNR* is marginally closer at the amino acid level to AtNIA2, while its exon–intron structure is reminiscent of the *AtNIA1* gene, showing three introns instead the single intron in *AtNR2/NIA2* (Fig. 3b). *Arabidopsis AtCDF1* represses *AtNIA2*, while activating *AtNIA1* (Varala *et al*., 2018), and in this regard, its repression by *StCDF1* identifies this gene as a *AtNIA2* functional ortholog.

To further support a function of StCDF1 in repressing the *StNR* promoter, while activating *StNPF3.1*, we performed transient transactivation assays in *N. benthamiana* leaves. Leaves were co‐infiltrated with *Agrobacterium* strains bearing the *35S:StCDF1.1* or *35S:StCDF1.2* (C‐end truncated) effector constructs and the *pStNPF3.1:iLUC* and *pStNR:iLUC* reporter genes (Figs S6, S7), and discs were collected 48 h after infiltration to measure LUC activity. Co‐expression of *StCDF1.1* or *StCDF1.2* was found in these assays to drastically suppress *StNR* expression levels, while enhancing *StNPF3.1* promoter activity (Fig. 3c). Moreover, whereas *StNR* repressive effects were independent of light, activation of *StNPF3.1* was significantly reduced in darkness (Fig. 3c), indicating that additional light regulated factors act together with StCDF1.1/StCDF1.2 to *StNPF3.1* promoter activation. We next examined whether StCDF1 transcriptional function is modulated by N availability, by placing the infiltrated discs on low‐N media. Notably, *StNR* repression was enhanced in low N, while this response was only observed in the light (Fig. 3d). On the other hand, activation of *StNPF3.1* was enhanced in low N and became independent of light (Fig. 3d). Together, these findings confirm that StCDF1 exerts an opposite transcriptional control on the *StNPF3.1* and *StNR* genes, while they indicate that StCDF1 transcriptional activity is modulated by N availability and the presence of light.

### *StCDF1* knock‐down improves performance of potato plants in low nitrogen

**Fig. 4:**
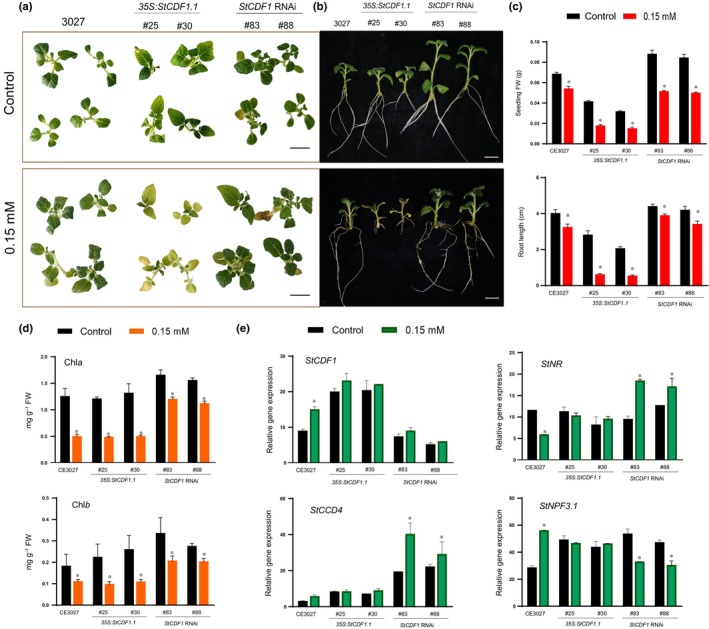
Effect of *StCDF1* expression on potato growth performance in low N. Phenotype of plants (a) aerial view, scale bar = 1 cm. (b) Side view of CE3027 (nontransformed controls), *35S:StCDF1.1* (lines #25, #30) and *StCDF1* RNAi (lines #83, #88) grown on MS and low nitrogen (0.15 mM) media, after 10 d of treatments, scale bar = 1 cm. (c) Measurements of the seedlings fresh biomass (g) and root length (cm) after 10 d of treatments. (d) Chl*a* and Chl*b* (mg g^−1^ FW) content of CE3027 (control), and the *StCDF1* RNAi and *35S:StCDF1.1* seedlings after 10 d of treatment. (e) Relative gene expression of *StCDF1*, *NITRATE REDUCTASE*, *NITRATE TRANSPORTER 1/PTR FAMILY 3.1 a*nd *CAROTENOID CLEAVAGE DIOXYGENASE 4* after 10 d low‐N treatment. Values are the mean ± SD. Asterisks indicate significant differences between control and nitrogen deficiency (0.15 mM) treatments for each genotype (*P* < 0.05), *t*‐test.

Direct StCDF1 repression of the *StNR* locus points to a central role of this factor in regulating nitrate utilization. We thus next investigated whether *StCDF1* expression was associated with growth defects in N deprivation conditions, by growing nontransformed CE3027, *35S:StCDF1.1*, and *StCDF1‐*RNAi transgenic plants on low‐nitrogen (0.15 mM) media (Fig. 4). We employed transgenic *35S:StCDF1.1* lines to these studies, as we had shown before that overexpression of the full‐length *StCDF1.1* and truncated *StCDF1*.2 alleles lead to functionally equivalent effects (Ramírez‐Gonzales *et al*., 2021). Regular MS medium, with a 60 mM total nitrogen content (Ramage & Williams, 2002), was used as optimal N media for comparison.

Remarkably, while all plants displayed an equivalent shoot biomass and root length on MS media, aside a trend of RNAi lines towards larger leaf folioles (Fig. 4a); growth phenotype of these plants was radically different in low N. RNAi transformants performed much better in these conditions, in contrast to the impaired growth of *35S:StCDF1.1* lines and the untransformed CE3027 control (Fig. 4a,b). Further phenotypic characterization of these plants showed that aerial growth and root length of RNAi lines was less suppressed in N‐limiting conditions than for WT or *StCDF1oe* plants (Fig. 4c). Chlorophyll content was also increased in the RNAi lines, consistently with these plants maintaining greener and healthier leaves in low N (Fig. 4a,d).

We further examined expression of the StCDF1 targets assessed for diurnal oscillation, to analyse whether changes in *StCDF1* levels affected their response to low N. Notably, *StCDF1* was found in these studies to be upregulated in N‐limiting conditions and, in line with this regulation, *StNR* transcript levels were reduced and those of *StNPF3.1* elevated on low N in CE3027 WT plants. Furthermore, consistently with our transactivation assays where low N was observed to boost StCDF1 transcriptional activity, higher expression levels of the *StNR*, *StCCD4*, *StWRKY41*, and *StNCED3* repression targets were observed in low N in the RNAi lines. By contrast, *StNPF3.1* expression was suppressed in these plants with respect to the WT and OE lines (Figs 4e, S3). These findings thus confirm that low N enhances StCDF1 transcriptional activity, while show that impaired nitrate reduction by *StNR* inhibition is to an important extent responsible for the shoot biomass and root growth defects in N‐limiting conditions of WT and *StCDF1.1oe* lines.

### Cultivar‐dependent allelic diversity in the *NR* promoter DAP‐seq region

Natural polymorphisms in the *StCDF1* locus were critical to expand potato cultivation into latitudes where autumns are chilly. *StCDF1.2* and *StCDF1.3* alleles, lacking the FKF1‐interacting domain, circumvent the strict short‐day control of wild *Solanum tuberosum* group *andigena* species, by stabilizing these factors that leads to suppressed expression of *StCOL*s and *SP5G* (Kloosterman *et al*., 2013). We here demonstrated that these alleles lead also to defective N utilization by suppressing *StNR* and other N‐related targets. Therefore, we conducted an *in‐silico* survey of the genomes of modern potato cultivars to search for *StNR* promoter polymorphisms that mapped within the StCDF1 DAP‐seq peak (Fig. S8). We used to this purpose early‐ and late‐maturing genotypes, already analysed for their *StCDF1* allelic composition (Campbell *et al*., 2010). Early *StCDF1.2*, *StCDF1.3*, and *StCDF1.4* alleles were for instance present in *Atlantic* and *Colomba*, while all copies in the late *Altus* and *Avenger* cultivars correspond to the WT *StCDF1.1* allele (Campbell *et al*., 2010; Hoopes *et al*., 2022).

Sequence alignment of the *StNR* promoters revealed that the DAP‐seq region includes five copies of the 5′‐YWAAAGRY‐3′ recognition motif. Notably, although all cultivars share highly identical promoter sequences, an inverted repeat is found directly after these motifs in the late cultivars (highlighted in Fig. S8). Moreover, *Altus* (late), *Castle Russet* (medium early), and *Spunta* (medium early) show a small insertion within this 269 bp region that corresponds to several copies of half the inverted repeat. This nucleotide repeat insertion is also found in *S. pinnatisectum* wild Mexican genotypes (Fig. S8), which shows is not a sequencing error. As such, genomic diversity within this regulatory region is greater than randomly expected, which suggests that promoter variants with lessened StCDF1 binding affinity were selected during recent potato breeding to counter‐balance inhibitory effects of early alleles.

### Impact of sequence polymorphisms and deletion of the DAP‐binding site on *StNR* promoter activity

**Fig. 5:**
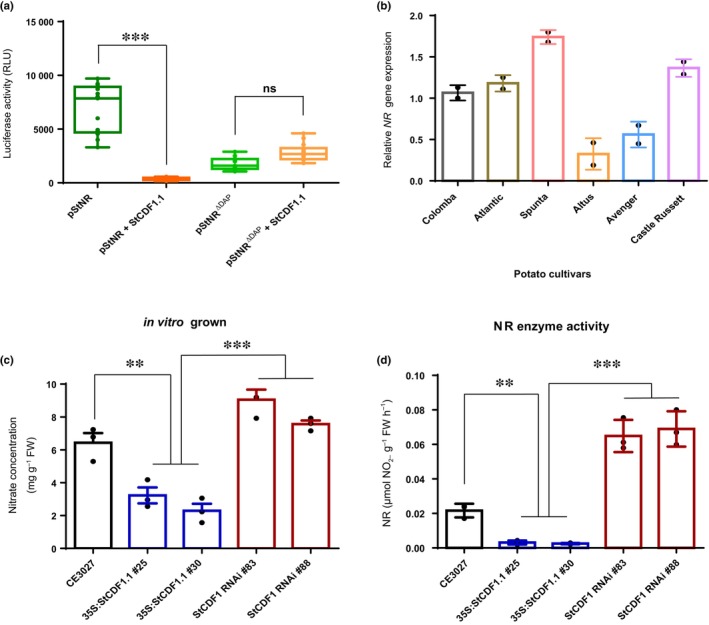
Deletion of *StCDF1*‐binding region on potato StNR promoter activity and impact of allelic variation on *NR* gene expression. (a) Transient transactivation assay using NR promoter with eliminated StCDF1‐binding elements (pStNR^ΔDAP^) shows no repression by StCDF1 compared with the intact promoter (*n* = 12), Boxplot depicts the median and interquartile range of luciferase activity. (b) Allelic variation in the StCDF1‐binding elements of *NITRATE REDUCTASE* promoter across different cultivars affect the expression levels of *StNR* gene. (c) Nitrate quantification (mg g^−1^ FW) of *in vitro* grown CE3027 (control), *35S:StCDF1.1* (line #25 and #30) and *StCDF1* RNAi (line #83 and #88) plants. (d) Nitrate reductase enzyme activity (μmol NO2‐ g‐1 FW h‐1) in CE3027, *35S:StCDF1.1* (line #25 and #30) and *StCDF1* RNAi (line #83 and #88) plants. Data represent the mean ± SD of *n* = 3 plants. Asterisks indicate significant differences for each genotype (*P* < 0.05), *t*‐test, ns, not significant.

We thus tested whether these polymorphisms had an impact on *StNR* transcript levels, by quantitative reverse transcription polymerase chain reaction analyses across the different cultivars. Reduced *StNR* expression levels were indeed observed in *Altus* and *Avenger*, showing six of the StCDF1 recognition elements vs the cultivars that lacked the more proximal motif (Fig. 5b), indicating that this additional site enhances StCDF1‐binding affinity. As such, this extra motif may have been maintained only in late cultivars, where reduced stability of the full‐length StCDF1 protein has a milder repressive effect on *StNR* expression (Fig. 5b).

To further confirm relevance of these elements in *StNR* transcriptional repression, we deleted all six motifs (pStNR^ΔDAP^) and tested this promoter construct in transient transactivation assays (Figs 5a, S9A). We included in these assays the *Arabidopsis* NLP7 effector construct described to activate the *Arabidopsis NIA1*/*NIA2* genes (Marchive *et al*., 2013), as a positive control. Notably, removal of the DAP‐seq region abolished repression by StCDF1 (Fig. 5a) but did not affect upregulated expression by AtNLP7, showing that this promoter construct includes all essential elements for activation by this N‐signalling regulator (Figs 5a, S9A,B).

We quantified endogenous nitrate levels (mg/g FW) of *in vitro*‐grown and soil‐grown plants (Figs 5c, S10), and against our initial prediction that increased *StNR* expression would reduce leaf nitrate content on its faster conversion into nitrite, we observed this to be elevated in *StCDF1* RNAi lines (line #83 and #88) as compared to *35S:StCDF1.1* (line #25 and #30) and CE3027 plants. Since NR is tightly regulated at the post‐transcriptional level (Lillo *et al*., 2004), we measured enzymatic activity to assess any protein feedback regulation. Enzyme activity was also elevated in *StCDF1* RNAi lines (Fig. 5d), suggesting that greater nitrate levels in shoots is caused by de‐repressed expression of other targets including several NRT1 nitrate transporters directly suppressed by StCDF1 (Fig. S11; Dataset S7). Hence, StCDF1‐silencing seems to favour nitrate uptake and transport to the leaves, in addition to promote StNR‐dependent nitrate to nitrite conversion, which altogether results in much better performance of RNAi lines in N‐limiting conditions.

## Discussion

Nitrogen fertilizers contribute to 2.3–2.7% of global greenhouse gas emissions (Eichner, 1990). Therefore, a major challenge of sustainable agriculture is to safeguard crops productivity while reducing environmental footprint of their cultivation. Nitrogen fertilization is critical in potato for high tuber yields (Tiwari *et al*., 2018). Nitrogen deficit leads to early senescence and chlorosis, decreasing tuber size, while the overuse of fertilizers suppresses tuber induction and delays tuber maturity (Li *et al*., 2016). Uneven nitrogen mineralization due to climate change (Masclaux‐Daubresse *et al*., 2010) has been predicted to strongly affect potato production in coming years. Here, through a combination of RNA‐seq and DAP‐seq studies, we provided evidence that StCDF1 not only acts as a canonical repressor of the day length tuberization pathway but also directly activates a broad group of genes involved in other biological processes. Further, we demonstrate that StCDF1 targets multiple nitrogen transport and assimilation genes, significantly affecting N homeostasis.

CDFs are clock outputs with a pivotal role in the day length tuberization pathway, while recent studies in *Arabidopsis* and tomato showed that members of this family modulate response to abiotic stresses, such as heat, cold, drought, or salinity (Fornara *et al*., 2015; Corrales *et al*., 2017). Here, we confirmed that StCDF1 directly regulates the potato *StCOLs*, *StCDF2*, and *StCDF3* genes, in addition to bind its own promoter and those of the *StCDF4* and *StCDF5* paralogs. In addition, StCDF1 was observed to directly regulate the *StBEL1*, *StBEL14*, and *KNOX1/STH20* homeobox genes, earlier shown to be expressed in phloem cells and their RNAs move from leaves to stolons (Mahajan *et al*., 2012; Sharma *et al*., 2014). Importantly, BEL1‐like family proteins in association with the POTH1 KNOX1‐homolog activate cytokinin biosynthesis and gibberellin catabolism in the stolons, more recent studies also showing that this complex regulates *SP6A* expression (Sharma *et al*., 2016). Our DAP‐seq studies thus underscore a role of StCDF1 in promoting tuberization beyond *StCOLs* regulation.

*Arabidopsis AtCDF1* (*StCDF2* homolog) had been identified as an early N‐starvation responsive TF sharing many common targets with the master nitrogen regulator NIN‐LIKE PROTEIN 7 (NLP7) (Varala *et al*., 2018; Alvarez *et al*., 2020) and remarkably we here show that StCDF1 regulates multiple N‐related genes (Dataset S4). Direct StCDF1‐regulated targets included a set of 160 N‐responsive genes (TargN‐RG) and found to be positively or negatively regulated by this factor. These comprised several nitrogen transporters and assimilation enzymes, in addition to NITRATE REDUCTASE (NR/NIA), that is negatively regulated by StCDF1. Genes encoding l‐asparaginase (*L‐ASNase*) and phenylalanine ammonia‐lyase (*PAL*) were likewise negatively regulated by this factor, but we were unable to amplify these transcripts by reverse transcription quantitative polymerase chain reaction.

Nitrate reductase encodes the first step in the inorganic nitrate utilization pathway, and its activity is required for subsequent synthesis of amino acids, proteins, Chl, and nucleotides, and normal plant growth and biomass accumulation (Liu *et al*., 2022). Consistent with such a key function, *StCDF1*‐RNAi lines, with derepressed *StNR* expression, performed better in N‐limiting conditions and displayed higher fresh biomass, Chl content, and root length than the WT (CE3027) and lines overexpressing *StCDF1*.

*NR* is encoded in potato, tomato, and pepper by a single genetic locus, whereas two *NR* loci are present in *Nicotiana*, presumably due to its allopolyploid origin (Yang *et al*., 2019). Green algae also typically contain a single *NR* gene, in contrast to the at least two *NR* gene copies found in most dicot and monocots (Berger *et al*., 2020). Potato StNR shares at the protein level higher similarity with AtNR2, but its intron–exon structure reminds the *Arabidopsis AtNR1* gene. *Arabidopsis* AtCDF1 positively regulates *AtNR1*, while negatively affects *AtNR2* expression (Varala *et al*., 2018). *AtNR2* is moreover expressed during dark hours (Olas & Wahl, 2019), in a similar fashion as the potato gene, which suggests potato *StNR* is the functional ortholog of *AtNR2*.

StCDF1‐dependent repression of the single *StNR* locus is predicted to have a significant impact on potato nitrate reduction. Here, we show that *StCDF1* is upregulated in N‐limiting conditions, while low N enhances its transcriptional activity, correlating with reduced *StNR* expression in the WT (CE3027). *StCDF1* RNAi lines performed better in low‐N media, suggesting that enhanced nitrate utilization due to derepressed *StNR* expression leads to the increased biomass and Chl content observed in these plants. Interestingly, these plants showed also elevated nitrate levels in leaves, while we predicted that faster nitrate to nitrite conversion would reduce shoot nitrate levels. We proved that NR enzyme activity was in fact elevated in StCDF1‐RNAi lines, supporting the notion that this effect is due to the concurrent upregulation of a wide range of nitrate transporters whose expression is regulated by StCDF1. Previous comparison of nitrogen usage efficiency (NUE) among commercial potato varieties evidenced that early‐maturing cultivars have generally lower NUE (Zebarth *et al*., 2003). Likewise, limited N had a stronger effect on tuber number and weight on commercial cultivars than in wild *andigena* species (Van Dingenen *et al*., 2019), consistently with stronger *StNR* repression by the StCDF1‐stabilizing alleles leading to defective N adaptive capacity.

**Fig. 6:**
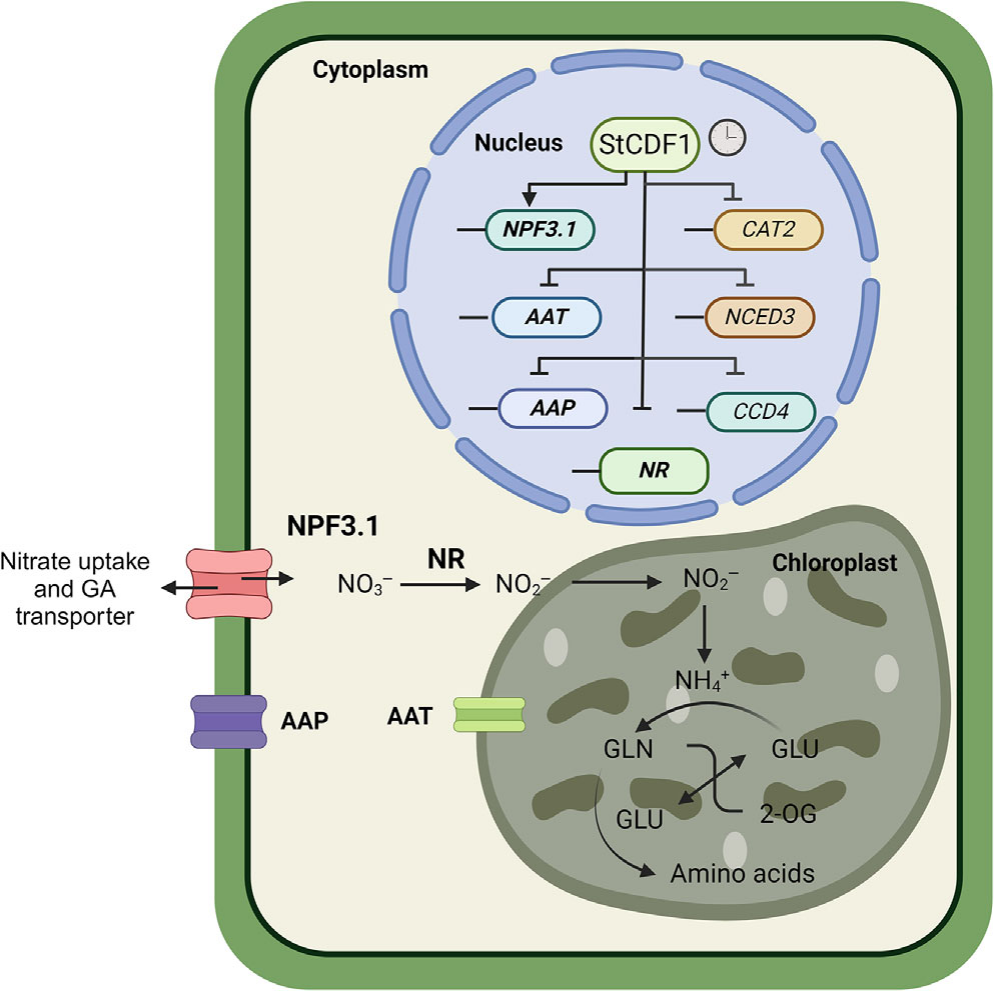
Molecular model for StCDF1 regulation of nitrogen‐related genes in potato. StCDF1 directly regulates a significant number of genes involved in nitrogen homeostasis and transport by binding to their promoter region. This transcription factor negatively (inverted T lines) and positively regulates (arrows) expression of these targets, presumably by acting in concert with other regulators. Suppression of the single potato *StNR* gene, encoding a limiting enzyme for inorganic nitrate utilization, indicates that the truncated alleles leading to stabilization of this clock output have a negative impact on nitrogen use efficiency (NUE). A complete list of its N‐related targets can be found in Supporting Information Dataset S5. *NPF3.1: NITRATE TRANSPORTER1/ PTR FAMILY 3.1; AAT: AMINO ACID TRANSPORTER; AAP: AMINO ACID PERMEASE; NCED3: 9‐CIS‐EPOXYCAROTENOID DIOXYGENASE 3; CCD4: CAROTENOID CLEAVAGE DIOXYGENASE 4. NR: NITRATE REDUCTASE. CAT2: CATALASE 2*. The clock on top indicates that StCDF1 and its targets are subject to circadian regulation.

On the other hand, StCDF1 directly activates expression of *StNPF3.1*, a low‐affinity nitrate *trans*porter upregulated in response to low N and with a dual function in the import of abscisic acid and bioactive gibberellins (GAs) into the endodermis of *Arabidopsis* roots and hypocotyls (David *et al*., 2016). Gibberellins negatively affect potato tuberization (Bou‐Torrent *et al*., 2011), and as such, it will be interesting in future studies to test the effect of nitrate supply on shoot and stolon GA levels. We speculate that *StNPF3.1* might delay tuber maturity through the transport of GAs into growing tubers, although additional studies are required to corroborate this hypothesis. Directly regulated targets of StCDF1, physiological function of NRTs, and a model on action of this clock output in regulating N homeostasis are shown in Figs 6 and S12.

Lastly, we identified several *StNR* promoter polymorphisms spanning the StCDF1 DAP‐seq region, which includes five StCDF1 recognition motifs. Notably, *Solanum tuberosum* group Phureja DM1‐3 and late potato cultivars show in this region an inverted repeat sequence that is absent in early cultivars. The forward half of this inverted repeat is moreover amplified in *Altus*, *Castle Russet*, and *Spunta* leading to a 269 bp insertion that is also present in wild Mexican *Solanum pinnatisectum* potatoes, resistant to late blight (Yang *et al*., 2017). Interestingly, we showed that *StNR* expression is reduced in late cultivars with the promoter hairpin structure, which suggests that these alleles were negatively selected in early cultivars as a compensation mechanism for StCDF1 stabilization. We further provided evidence that deletion of the entire DAP‐seq region suppresses StCDF1‐mediated inhibition of the StNR^ΔDAP^ reporter, whereas it is of no significant effect on NLP7‐dependent activation of this promoter construct. Therefore, selection of *StNR* promoter variants missing the promoter hairpin, together with gene editing approaches to delete the DAP‐Seq promoter region, emerge as excellent tools to improve nitrate reduction efficiency of elite potato cultivars. The generation of such *StNR‐*based cultivars can provide a tool to reduce the environmental footprint of this world‐wide cultivated crop and boosting its productivity.

## Competing interests

None declared.

## Author contributions

SP and CWB designed the experiments. MAS and LRG performed the experiments, analysed the data and assembled the initial text manuscript and Figs MAS generated the promoter reporter constructs and conducted all the transactivation assays. ES and JMF‐Z carried out the DAP‐seq experiment, and JMF‐Z analysed the NGS results. MO performed the time course reverse transcription quantitative polymerase chain reaction. CWB and SP supervised the work, and along with MAS, critically edited the manuscript. MAS and LRG contributed equally to this work.

## Supporting information


**Dataset S1** Significant DAP‐seq peaks in the three experiments.

**Dataset S2** RNA‐seq results from *StCDF1* transgenic plants.

**Dataset S3** Heatmap of StCDF1 target genes and binding sites peaks (5 kb upstream of the TSS) as obtained from DAP‐Seq and RNA‐Seq data.

**Dataset S4** Gene Ontology term enrichment analysis of StCDF1 targets.

**Dataset S5** StCDF1 target genes related to nitrogen response.

**Dataset S6** Oligonucleotides used in this study.

**Dataset S7** Differentially expressed nitrate transporters in StCDF1ox vs StCDF1rnai RNA‐seq data set.

**Fig. S1** Overview of DAP‐seq analyses.
**Fig. S2** StCDF1 binds the promoters of genes related to potato tuberization.
**Fig. S3** Potato StCDF1 downregulates *WRKY* transcription factors.
**Fig. S4** Phylogenetic tree of *NITRATE REDUCTASE* from *Arabidopsis* and *Solanaceae* plants.
**Fig. S5** Sequence alignment of the NITRATE REDUCTASE proteins from *Arabidopsis* and *Solanaceae*.
**Fig. S6** *NITRATE REDUCTASE* putative promoter sequence.
**Fig. S7** *NITRATE TRANSPORTER 1/PTR FAMILY 3.1* (*NPF3.1*) putative promoter sequence.
**Fig. S8** Allelic variation of the potato *NITRATE REDUCTASE* promoter.
**Fig. S9** Removal of StCDF1‐binding elements reverses the StCDF1.1 repression of NR/NIA1.
**Fig. S10** Nitrate quantification (mg g^−1^ FW) of soil‐grown potato plants.
**Fig. S11** Potato nitrate transporters differentially expressed in *StCDF1ox* vs *StCDF1rnai* RNA‐seq data set.
**Fig. S12** Physiological function of nitrate transporters.
Please note: Wiley is not responsible for the content or functionality of any Supporting Information supplied by the authors. Any queries (other than missing material) should be directed to the *New Phytologist* Central Office.

## Data Availability

The data that support the findings of this study are openly available in ‘Gene Expression Omnibus‐NCBI’ at https://www.ncbi.nlm.nih.gov/geo (GSE203123, GSE203124, and GSE203114).
