# Supplementary material for: StCDF1: A ‘jack of all trades’ clock output with a central role in regulating potato nitrate reduction activity
Source: New Phytol. 2024 Nov 6;245(1):282–98. doi: 10.1111/nph.20186 (PMC11617646; doi:10.1111/nph.20186)
Supplement: Supplementary file 8 — Fig. S1 Overview of DAP‐seq analyses. Fig. S2 StCDF1 binds the promoters of genes related to potato tuberization. Fig. S3 Potato StCDF1 downregulates WRKY transcription factors. Fig. S4 Phylogenetic tree of NITRATE REDUCTASE from Arabidopsis and Solanaceae plants. Fig. S5 Sequence alignment of the NITRATE REDUCTASE proteins from Arabidopsis and Solanaceae. Fig. S6 NITRATE REDUCTASE putative promoter sequence. Fig. S7 NITRATE TRANSPORTER 1/PTR FAMILY 3.1 (NPF3.1) putative promoter sequence. Fig. S8 Allelic variation of the potato NITRATE REDUCTASE promoter. Fig. S9 Removal of StCDF1‐binding elements reverses the StCDF1.1 repression of NR/NIA1. Fig. S10 Nitrate quantification (mg g−1 FW) of soil‐grown potato plants. Fig. S11 Potato nitrate transporters differentially expressed in StCDF1ox vs StCDF1rnai RNA‐seq data set. Fig. S12 Physiological function of nitrate transporters. Please note: Wiley is not responsible for the content or functionality of any Supporting Information supplied by the authors. Any queries (other than missing material) should be directed to the New Phytologist Central Office. [file NPH-245-282-s003.docx]

***New Phytologist* Supporting Information**

StCDF1: A*" jack of all trades”* clock output with a central role in regulating potato nitrate reduction activity

orena Ram


ırez Gonzales

1

, Li Shi

1

, Sara Bergonzi Bergonzi

1

, Marian Oortwijn

1

, Jos


e M. Franco-Zorrilla

3

,

Roberto Solano-Tavira

3

, Richard G. F. Visser

1

, Jos


e A. Abelenda

2,†

and Christian W. B. Bachem

1,*,†

1

Plant Breeding, Wageningen University & Research, PO Box 386, Wageningen 6700 AJ, the Netherlands,

2

Centro de Biotecnolog


ıa y Gen


omica de Plantas, Universidad Polit


ecnica de Madrid (UPM), Instituto Nacional de

Investigaci


on y Tecnolog


ıa Agraria y Alimentaria (INIA), Madrid 28040, Spain, and

3

Departamento de Gen


etica Molecular de Plantas, Centro Nacional de Biotecnolog


ıa – CSIC, Madrid 28049, Spain

**Maroof Ahmed Shaikh^1 ♱^, Lorena Ramírez-Gonzales^2,4 ♱^, José M. Franco-Zorrilla^3^, Evyatar Steiner^3, 5^, Marian Oortwijn^2^, Christian W.B. Bachem^2, 6*^, Salomé Prat^1*^**

Corresponding authors: Salomé Prat and Christian Bachem

Email: [*salome.prat@cragenomica.es*](mailto:salome.prat@cragenomica.es) and [*christian.bachem@solynta.com*](mailto:christian.bachem@solynta.com%20)

Article acceptance date: 21 September 2024

**This PDF includes**

Figs. S1 to S12

Other supplementary materials for this manuscript include the following:

**Dataset S1**. Significant DAP-Seq peaks in the three experiments.

**Dataset S2.** RNA-Seq results from *StCDF1* transgenic plants.

**Dataset S3.** Heatmap of StCDF1 target genes and binding sites peaks (5 kb upstream the TSS) as obtained from DAP-Seq and RNA-Seq data.

**Dataset S4.** GO term enrichment analysis of StCDF1 targets.

**Dataset S5**. StCDF1 target genes related to nitrogen response.

**Dataset S6.** Oligonucleotides used in this study.

**Dataset S7.** Differentially expressed nitrate transporters in StCDF1ox vs StCDF1rnai RNAseq dataset.


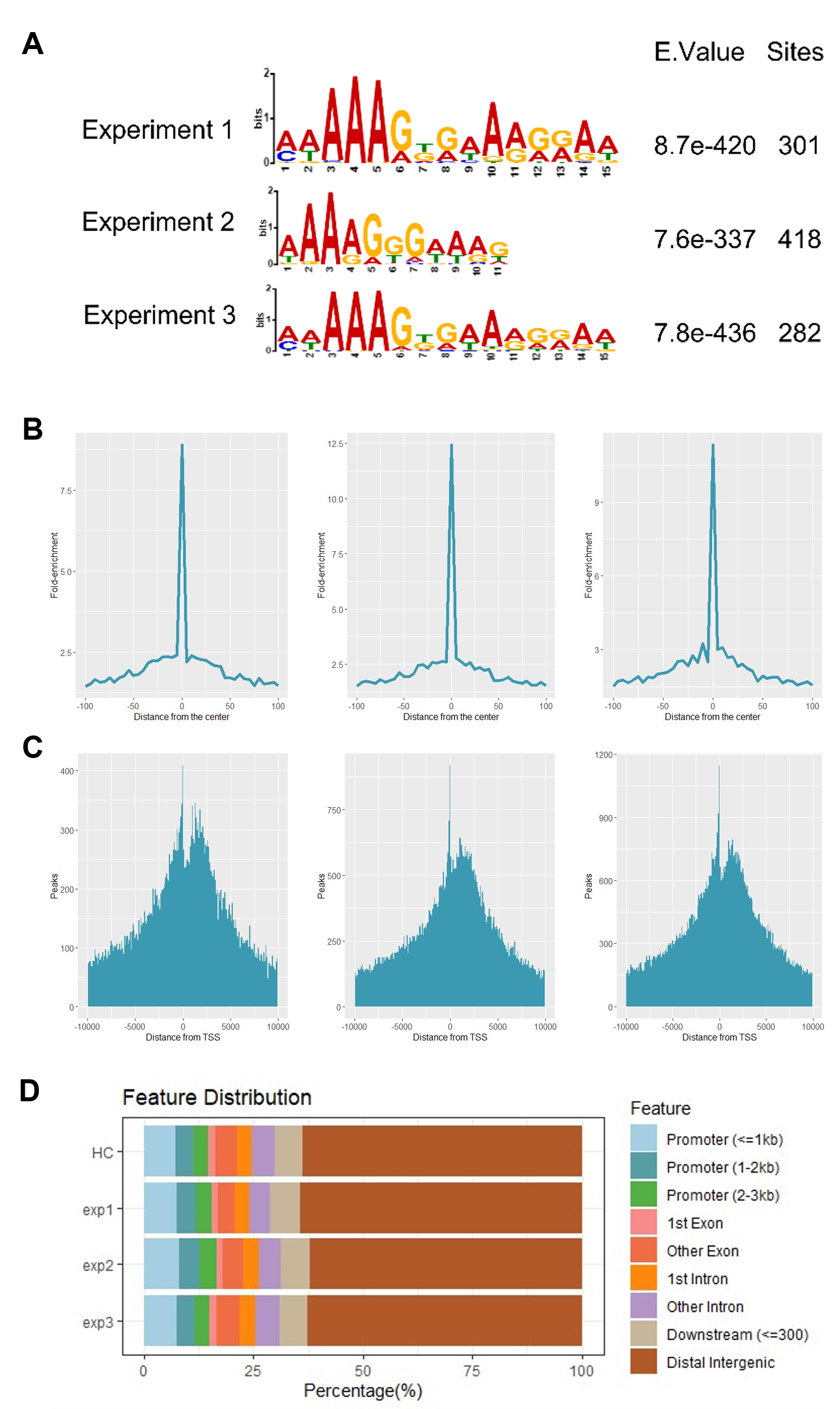


**Fig. S1. Overview of DAP-Seq analyses.**  **(A)** Consensus sequence for potato StCDF1 binding generated from the 600 more significant peaks in three independent experiments. **(B)** The StCDF1 binding site motif (5’ WAAAG 3’, W= A or T) was found in all three experiments to be enriched at the center of the peaks. **(C)** Enrichment of DAP-seq peaks in the proximal promoter regions near to the TSS. **(D)** Overall distribution (in percentage) across genome features of the StCDF1-binding peaks obtained in each of the experiments, and its comparison with the “high confidence” peaks detected in at least two experiments.

**
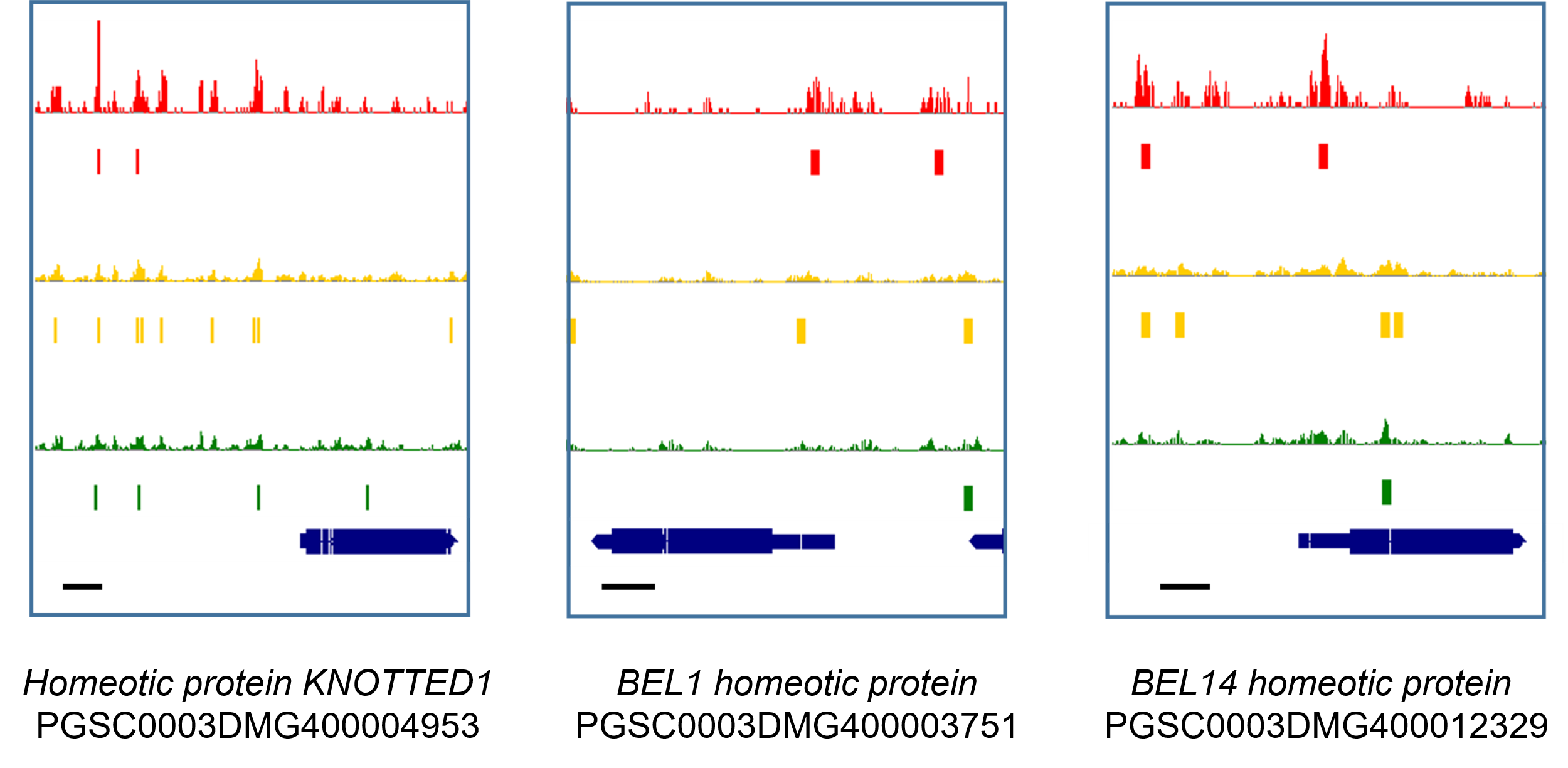

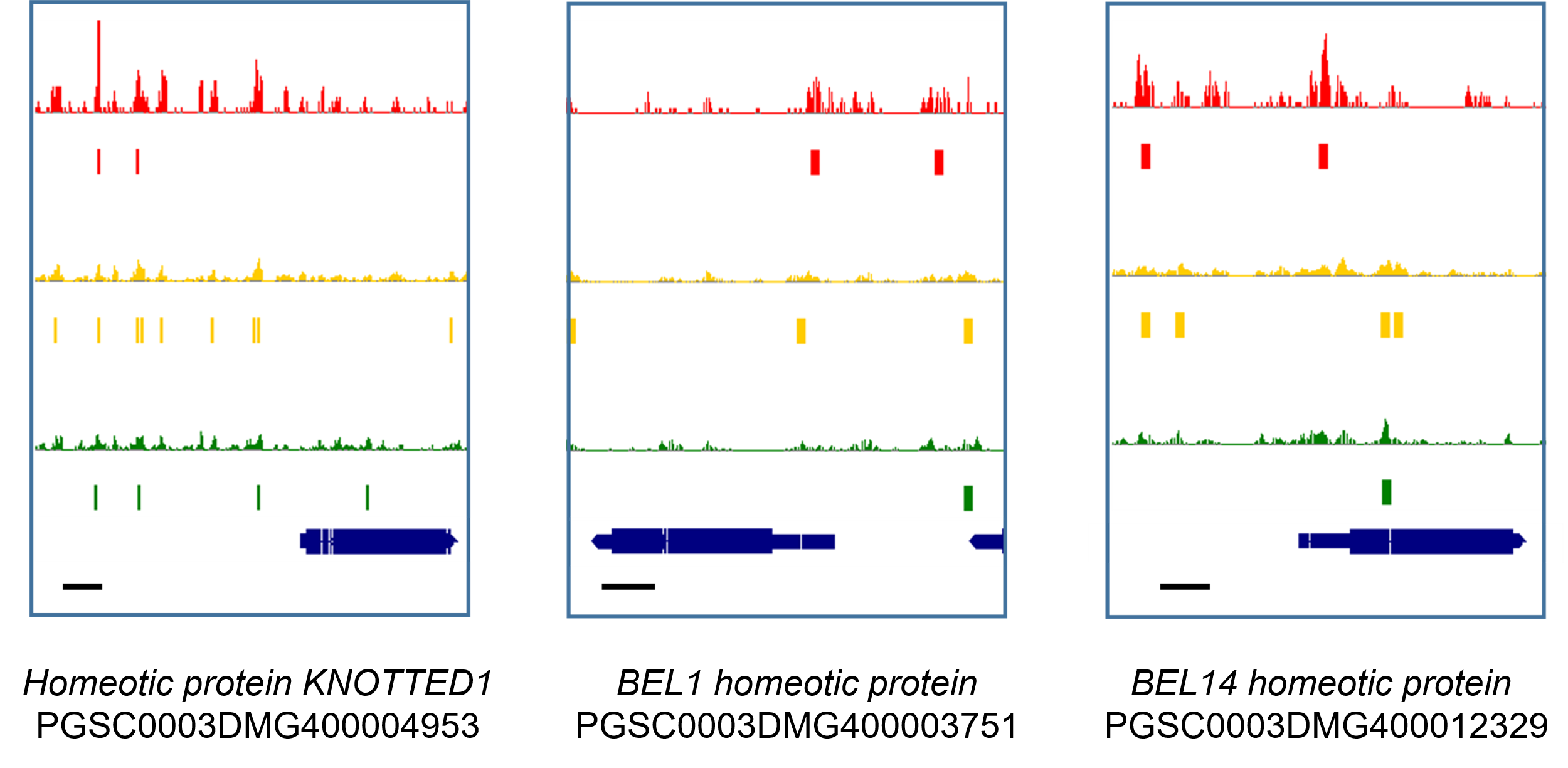

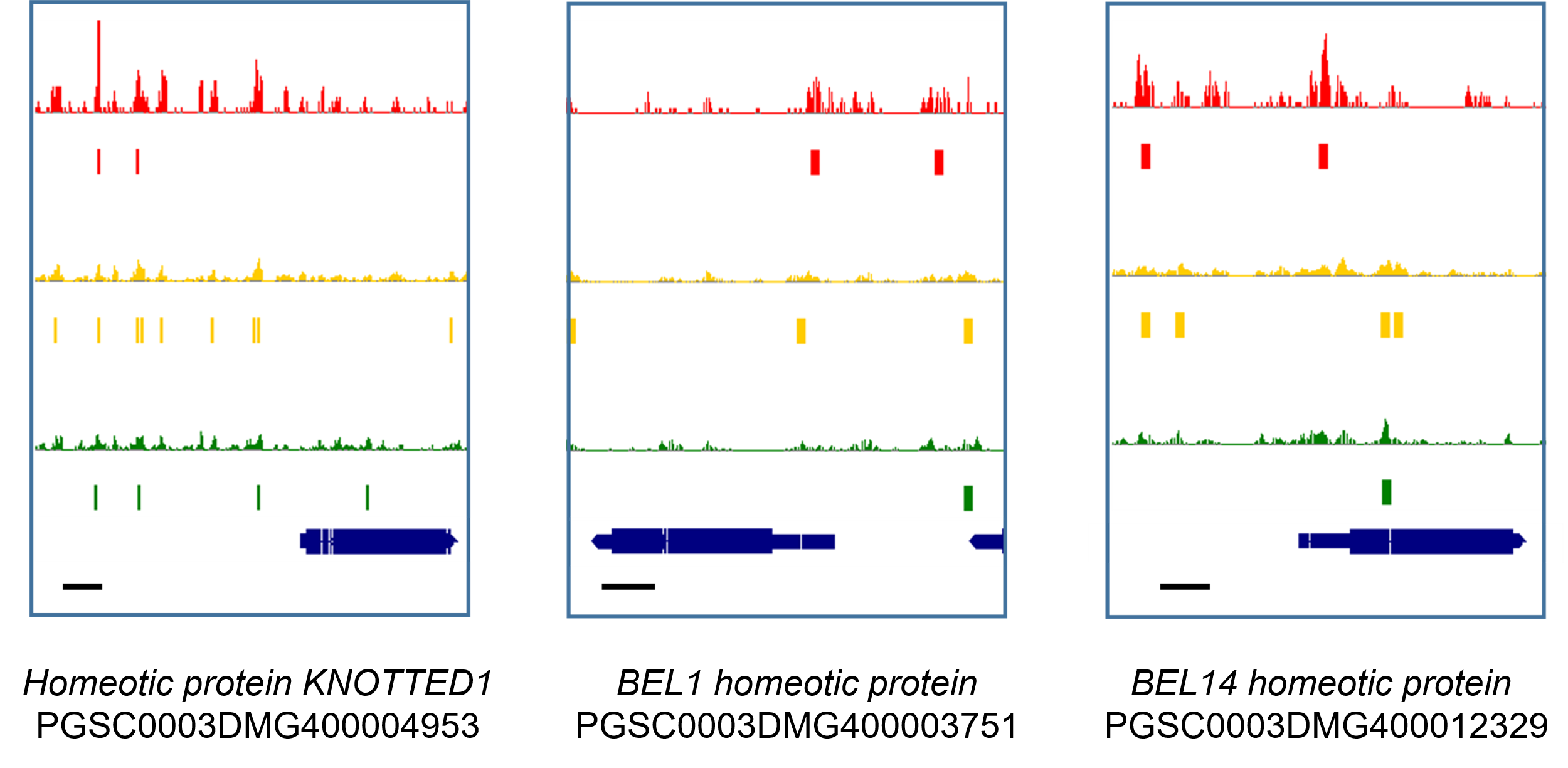
**

**
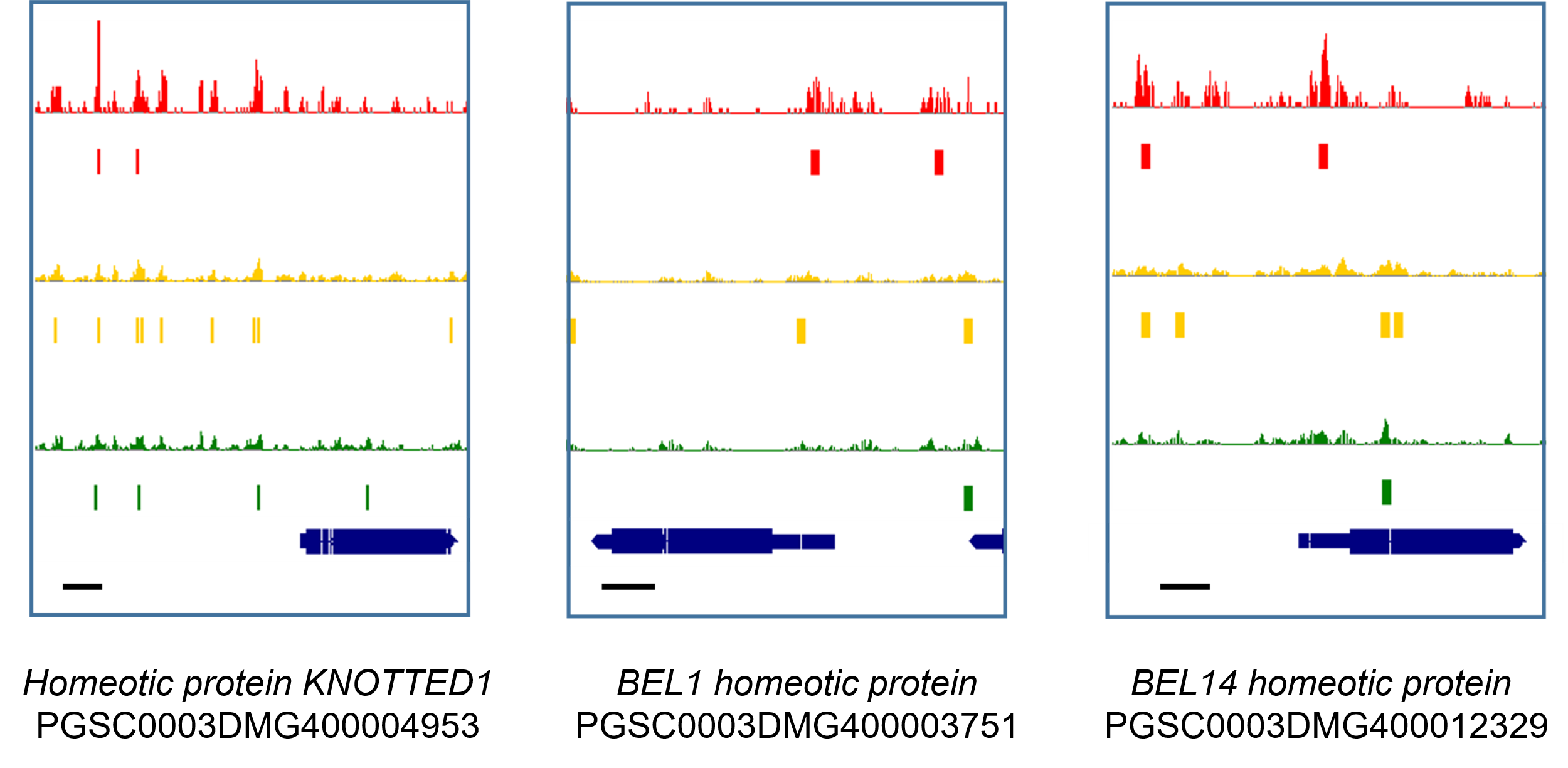
**

**Fig. S2.** **StCDF1 binds the promoters of genes related to potato tuberization**. DAP-Seq binding peaks of StCDF1 in the promoter regions of *BEL1 homeotic protein (BEL32), BEL14* and *homeotic protein knotted-1* (*KNOX)* genes. The diagram shows the enriched regions obtained in experiment 1 (red bar chart), experiment 2 (yellow bar chart), and experiment 3 (green bar chart). Below the bar charts are the significant binding sites identified after statistical analyses. Genes are represented at the bottom in blue (For more details, see Methods). Scales represent 1 kb.

**Fig. S3.** **Potato StCDF1 downregulates *WRKY* transcription factors.** Four-week-old plants (CE3027 un-transformed controls, *StCDF1.2oe* and *StCDF1* knock-down lines) were used for RT-qPCR analyses. Values are the mean ± SE of two biological replicates. Sequence of primers used for *WRKY41* (PGSC0003DMG400000211) and *WRKY50* (PGSC0003DMG400008188) amplification is given in Dataset S6.


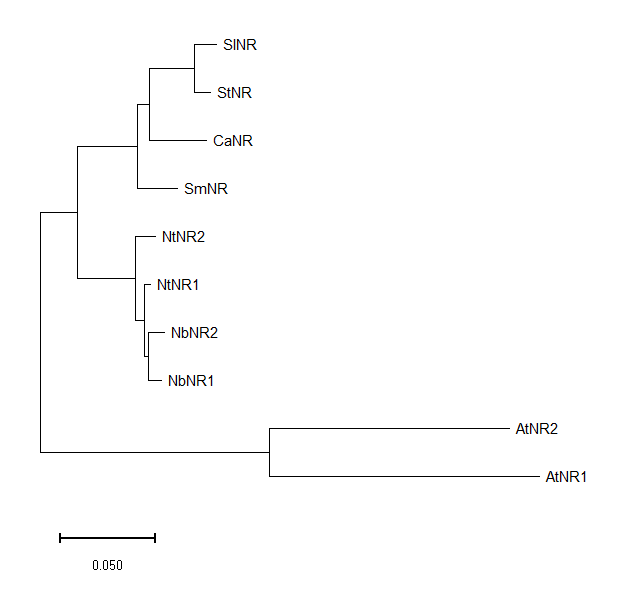


**Fig. S4.** **Phylogenetic tree of *NITRATE REDUCTASE* from *Arabidopsis* and *Solanaceae* plants.**

Phylogenetic tree was generated by Maximum Likelihood method using Arabidopsis (AtNR1 and AtNR2), *Solanum tuberosum* (StNR), *Solanum lycopersicum* (SlNR), *Nicotiana benthamiana* (NbNR1 and NbNR2), *Nicotiana tabacum* (NtNR1 and NtNR2), *Capsicum annum* (CaNR) and *Solanum melongena* (SmNR) full-length amino acid sequences as query in the Mega11 software.


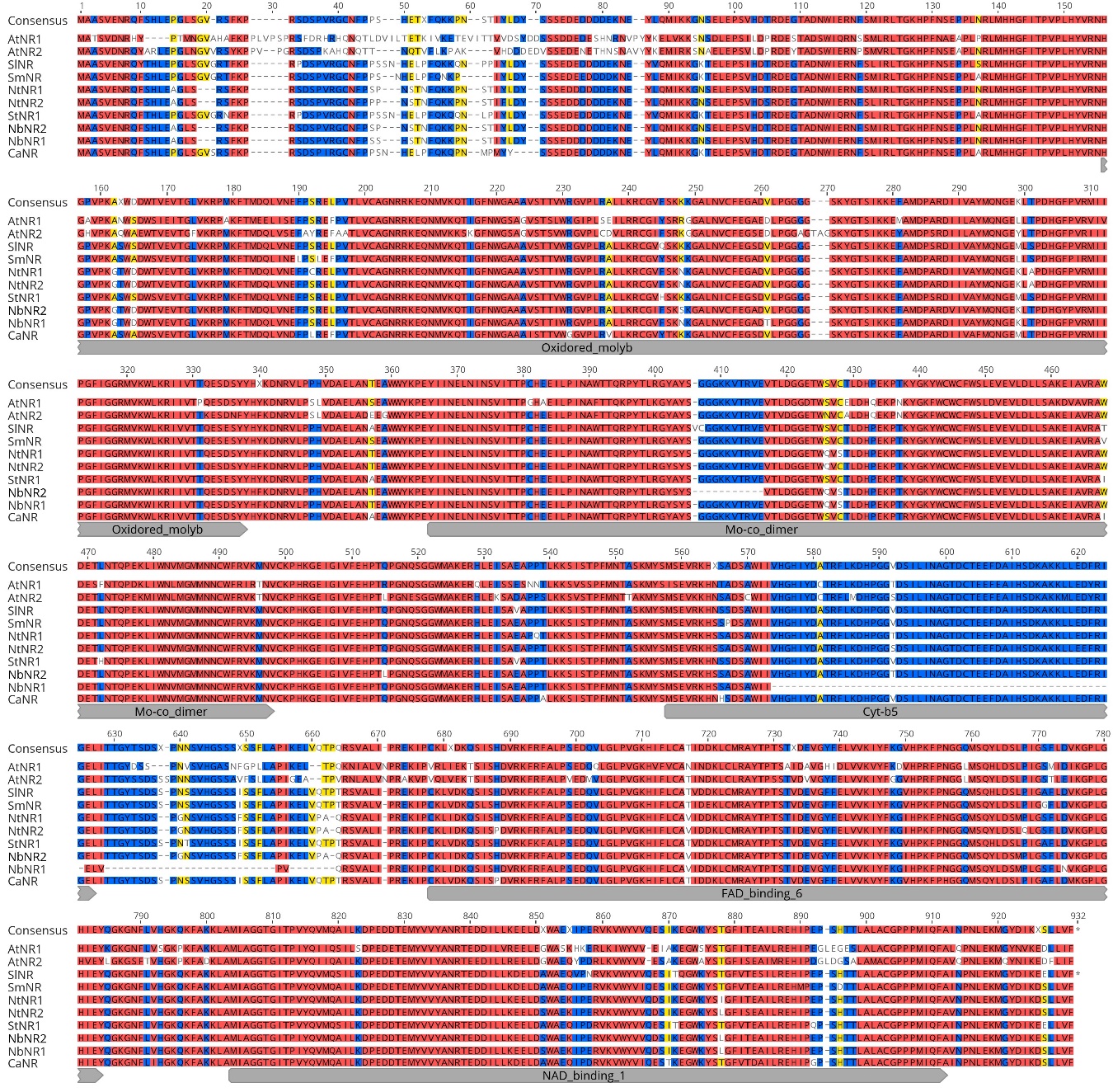


**Fig. S5.** **Sequence alignment of the NITRATE REDUCTASE proteins from *Arabidopsis* and *Solanaceae*.**

The protein sequences of Arabidopsis (AtNR1 and AtNR2), *Solanum tuberosum* group Phureja DM-1-3 (StNR1), *Solanum lycopersicum* (StNR1), *Nicotiana benthamiana* (NbNR1 and NbNR2), *Nicotiana tabacum* (NtNR1, NtNR2), *Capsicum annum* (CaNR1) and *Solanum melongena* (SnNR1) were extracted from NCBI database. Amino acids with high conservation and low conservation are shown in red and blue, respectively. The phosphorylation site at S532 is marked with a green rectangular box. The grey boxes below the sequence indicate the Oxidored_molyb, Mo-Co_dimer, Cyt-b5, FAD_binding_6 and NAD_binding_1 functional domains.

>chr11:10182740-10187159(**promoter *NITRATE REDUCTASE***)
GATCTGATCAAATCGATCGTTACACAAAATAAGACTTGTCATCATTTATAGAATTAACGATGCAATAGAATAAAATTGAAGCAATAATCAAAACAAACATTATATTTAAATTAACAATACAATAAAAATATT

TTTTTAATACAGTAGACAAGTAGTGGAAATATAATTATACGTACATTTA**GGTAGGTAACTAACTAGTGG**AGGCATCATGTGGTTTAAGATTTACGAAGACATAATAATCCAACATTAGATTATGTTTGTTGAACGCGACGAAGTTTCATTGCATCTGCTAATTATACGCATTTGCACACGTACCCAGAATCGCTAATCTTAATGCGGATAATTAACCCATAATTCAAAATGGATTAAGAGCCCGTTTGGATTGATTTTTAAAAAAAAATAGTTTTTAAGTCAAAAATAAAAAGTCAAACTGAAATTACTTTTAAGTCAAAAAATAAAAAGTTGGGGGGACCTACTTTTGGTTTTTGACTTATTTTAAGTCATTTTTTACCTTACTAAACACTTCCTAACTTATTTTAAGTTATTTTTTATATTTGTCAAATACTTTTAGAAGTCAAAAATTGACTTAAAAGTTCTGAGGGTGCTTATTTTGTAGGGTTTAAGAAAAACAATAGTACTACCTAAGGGTGTTCACGGTTTGGATAAAAACCAATCCAAACAGAAAAACCAAACCAAACCGATAAAATAAAACCGATTTTATTTGGGTTTGGTTTTAGATTTTTGAAAACCAGTAGTATTTGGTTTGATTATGATTTTATTTGAAAAAACCGAAGAAATAACCGAACCGATAAATTATATACATAAATTTTATTAATATACATACTTAATATATTGTTTTATAAACAATTTTGAAGATCTTATATATGTTTTCATTAAAATTTCCTTATACTTTACTTATTGAAAGCAAGAATGCCCAAGGTGAAAACATTTATCTTATTGATTTCATGTATATGAGTGTCTATGACAATTCTATGTGAGACTGAAAATGTTATTGTCTCTTCATTCTAGGCCAATATAGTGAATTTTAAAGCTTTATTGATAGTAAATTCAGTGATCGAAAGTTCGGTAATTTAAATCATTCTAACTATTTTTTGTTTTGAATGGATTGTTGTCACTTTTTTCACATTTTGAATGAATTGTTTCTTTTATTTGTGTATGGTTAATAACCAAACCAAACCAAACCGATAAATGTATATTATATTTGGTTTGATTTGGTTTTGATAATTTTAAAACCGATTAAGTTGGTTTGGTTTTGATTTTGACCAATAACCGATCCAAACCGACCCGTGAACACCCCTAGTACTACCTATGGATAAATAATTATTCATGAAATATATATATATATATATATATATATATATAAATAATTACACCTGGAAAATATGTATGAATGCCATGTAAATAAAAATGAAAAAGTATTTACACAAATATGCCTAGTTGAAGTGGAAATTTTATAATGGTAATTCTCCAAGAACCTTTAAAAAAACGTATTTGAAATTTGAATGGATAAGGGAAAAATCTCATAAAGAAATTTTAAAATTGAGAGGCGAGGGGTGAAATTATAATTTTTGTTAGATTGAGTAAATTTTTACTGAAACAAAACATGTTATAAACCTATAACAATTTTCGGTAGTTTTGATATTTGGAAATTGTAGCAATCGTTATGTTTAAGTGTTATTGAAAAGGTTGTTCCTGTTATCGAAGGATGTGGTGCAGACGTGCAGTTTTGATTTTTCTTGATTTTAAATATTGGATAGTAATTTTTTTGATAGAGAGCGGCAATCGTTACTTTCGAATGAGCCTTATTCGACACAAATTTAAATTAATCGAGTTTCAATTTGTATATCAAAACCAAATAAGAAACTAATTAGTTCAATTAGTTGGCTGACTAAACTTTCACCTTATTGGTAACGATACAATTTCTCCCTTATAATCTCCTCCACAAATTTGCAATTAGCATATTGCAAGTCCATTTCAGATTGAAATAGTTCGTTATAACTAACAATATCAAAGAAAACTTTTGAATGTGCTTGCTAACAAAAATATCACTACTATCTTATTTTTGGCTTAATACATTTACGATTCTTTAAACTTGTTAATAAATTTCATATAAATATTCAAATTACGACTTGTTCCCATCAATAAACCTAAACACATAATACCATAGACACTTTCAATTTATTTTTTACAAAAAAAAAATATGAAGGTATAGTTAATGAAAAACATGTAAGGCTTGTTTTACACATAGGCCATAAGGTCATGAGTACCATTTTTACCCAAAAAAATTTAGTATGTAGTGGTAGTAGCTGTGGAGCCGTTAATAATGTTTATTGTGACTCAAAACCACGGCAAAAAAAACAAGAAACAGCTGTTAGCCCTAACCAACAGCTGCATATATTTAAGCCATTTCAGAAACAACACCCCTTTTTCTCACTGACCCCAGCCGCGTTAAAAGTGGATCAAGTATCGAAAACACTCTTAAATTCAATGTAAATTATTAGTTTTTTTATTTTTGAATTATTAATATTTTAAGAACATCTTTTACTTGACTAATTTAACTTAAATAGATCTCATACACCCTTAAATTTCCGTCAAGCTTAGGATGTTGTTAACATTTTTCTCAACTTTTTATTAAGAGTTTCATGCTTATACAAGAGTAATTTGTTTGAGACTGCTTGTTGTATCATGTGACAGATTGAGAGTGTATCTAAATTTGATTGGTCAAGTAAAAGATTGAAACTAATAATTTACATCAAATTTAGAATTGTTTTTAACACTTCACGTCGTAAAATTGTAAAGTATAGACCAATTTATATTTTTGCGGATGATGTCACAAACTTTTGTAATCAAAATTCAATTTTGGTGTCGATTTTTTGGGTCCTACTTATGTATAGTAAATATGGGGTTGAGACAGTTCGCAACGTTTCTATATAAACACAACTCTCCGCACTCACAATATTCGTTCCCAAACCAAACTAAGAACAACCAAAATCTAAATACAAAGAAAGAAAAATCTCTTTTCTTTCTTCTTTCTTTTCTATTTTTCCTAAATTTTCCCATTCATATATATATATTTTTAAT

Fw: prom*StNR*

Rv: prom*StNR*

TTCTCAATAA**TCAATGGCGGCATCTGTTG**AAAACAGGCAGTTCACTCACCTTGAACCGGGTTTATCAGGCGTCGGCCGTAATTTCAAGCCTAGGCCTGATTCCCCGGTTCGTGGTTGCAACTTCCCTCCGTCATCCAACCATGAACTCCCTTTCCAAAAACAACAAAATCTCCCCATTTACCTTGATTACTCGTCTAG

**Fig. S6. *NITRATE REDUCTASE* putative promoter sequence.**

The nucleotide sequence (PGSC0003DMG400030212) was extracted from potato DM v4.04. In Grey is indicated the peak sequence obtained from the DAP-Seq data, Blue box: experiment 2, Black box: experiment 3. In bold are the sequences of the primers used to clone the promoter into the luciferase vector. ATG: Start methionine.

>chr06:8721597-8728300 (**promoter *NITRATE TRANSPORTER 1/PTR FAMILY 3.1***) NPF3.1

CTGGTTTAAGACTATCTTTTATTAATACTCTTTAATTTTATATATTGTGATTTTTATTTTTTATTTTTGTAAAATCCGTCATCTGAAGGTTTTTTTTTTTTTTTTTTTTTTGCAAATAAACAAAAAAAGAAGAAGAAAAACTAACCTCTCAAGGTTGGATTTTGCATTTTTTTTTTTAAAAAAAACTGACTTTAGAAG

GTTGGTTTTTGTAATCTTTTTTAAAGTCGATTGACATTTT**TTTCGATTTGGGCGCTAAAAA**ACCTAACCTTCGGAGATTATTTTTGACAATTTTTTAACTGTAAGCTACTTTTAATCAGCTAATGCAAACAAAATCTACAGAATAACCCAATGACCATTTTTGGTAAGATTTAAAAACTAAAACATGTAGATAAACTTTGTTTAACATGTTATTGTCTTTTCTCCAGAACAATTTCTGCAGATTCTAATCCAACTAGGTAATGCAAATTTGATTTAATAGTCACATGTACATCTACATTCATGAGCTGCAAAGTGAAACAAAAAACTGCAAATGGGACCCCACAATACCGGCCTGTATTACTCACTCTAGGGGCGGATCTACCCACAATGGTTAAAATCTAGGAAAATCTATTTGTTGCTCAAGTTATTATCATATATTAAATTTAAATCACATTAGAATTTTGAACGAGTATTTGATTATAAATTTTTAATGTGTTATATCATAATCCTAATATATATTTCACTTCTATTTTGAAAAATATGAACGTGACTTATAAACATAAATTTTAAAAACTATTAAGAATGTCCAATCATACCAAAACAAACATATTTTCTTATTCCATATTATGTAGAGCCTTCATCGATGTCGTGCATTATTATTATTATCACAAATCATATATAATTCTATATATAGATAAGTTTGACACAAATTATCATTTGAATAACAAATTCTAATAACCAATCACTAGTAACTACAAATTGTTGCTTCCTGTATTCAACATAGTACTAAACCAGAAAACTATTAAGATGAAAAGAAATAAAAATATTTGAGGCCATAGAATTTACTGTTATATTATGATAGCCAACGAGACCGAAAATACAACAATGATTTGTTTTATGGACAGTTATATTACCAGCCCAAATGAGGTAGTGCCTAATGTCACCGGGAGCGAGTTGGTAGGGGGGGCCTGAGATCTTGTGCCTTTAAGAAATTTAATCCCTTCACTTTATTTGTGTTTATGGATTATTTCCTCTCATGATGAAATGGATGACCTATTAAAGAAGTAGCAGTACCTCAAACTTCAATAATTGTAGTGAACTGAAAAGGCAGCAATGAAATTACACAAAAAAACTCTACTTTTTTTGTAAGAAAATGTATGCAGAAAGAATAAAATGTATGCAGAAGGAAGATGAATTGATTTTGAAAAACGAGAGAAAATTCCTCAATTTATAGTTAACAAAGGGTAGTATGAAAATGTGCTAATTGTGTCTTATCGGAGAGGTCACAACCCTTTCACAAAAGAGACAACATTATAGAAAGGTCACAACCCTTTGGAAAATGCACAATCTTTTAGAATGATCACAATCCTTTAGAAAAGACACAATCTTTCAAAAAGGTCACAACCTTTTATAGAGTCACTCCCTTTCAAAAGAACCACGACCGTTCATTTTCCATTCAAACCTTTAAAAACCAACACAAATTAGTTACAAAAATCCATCAGGGCAAAAGAAAACAATAATAATACTAATATACTAACAATAGTAAAAGTTTGAGGGATATATATATATATATATATATATATTTTTTTTTTTTTTTTTTTTGATAAAATACAAAAGTTTGGAGTAGATTTTAAAATAAACAAACAATTATATTTTGAGCAAAACAAAAGAATCTGAAAAAGATGTTAAAATTTAATATTTTGATTTCCAAAATCTCTCAAGTAGTAAGATAAATTCTATGACCATACATGTATTTTTTTACCAAAAAATACTTTAATACCTCTCCATTTAATCAAACTTACTCAAGTAGGGAGAGAAGCAAAAAAGGTGACCAAATACTTCCAAATGAAAACCAATGGATGAGACAAATTAACATGGGGCTAAGAATGTCAATAAATATCCCCTACATCTATAGGGAATTTCTCAAAATTGCTTGCATTTCATAACTAAGAGTAGCTAGTCACG**ATG**AGTGAAAATATGAAGGTGGCCAAGCAAGAGCTAGTTGTTCAAGAAAATGGCAAGAAAGAAGAAGATGAGACAA

TTGAAACTGAAAAAAGGAAGCT**TGGAGGAATGAAAACAATGCCC**TTTATACTTGGTAAGTTA

Rev: prom*StNPF3.1*

Fw: prom*StNPF3.1*

**Fig. S7.** ***NITRATE TRANSPORTER 1/PTR FAMILY 3.1 (NPF3.1)* putative promoter sequence.**

The nucleotide sequence (PGSC0003DMG400025395) was extracted from potato DM v4.04. In grey is indicated the peak sequence obtained from the DAP-Seq data. Blue box: experiment 2, Black box: experiment 3. In bold are the primers used to clone the promoter into the luciferase vector. ATG: Start methionine.


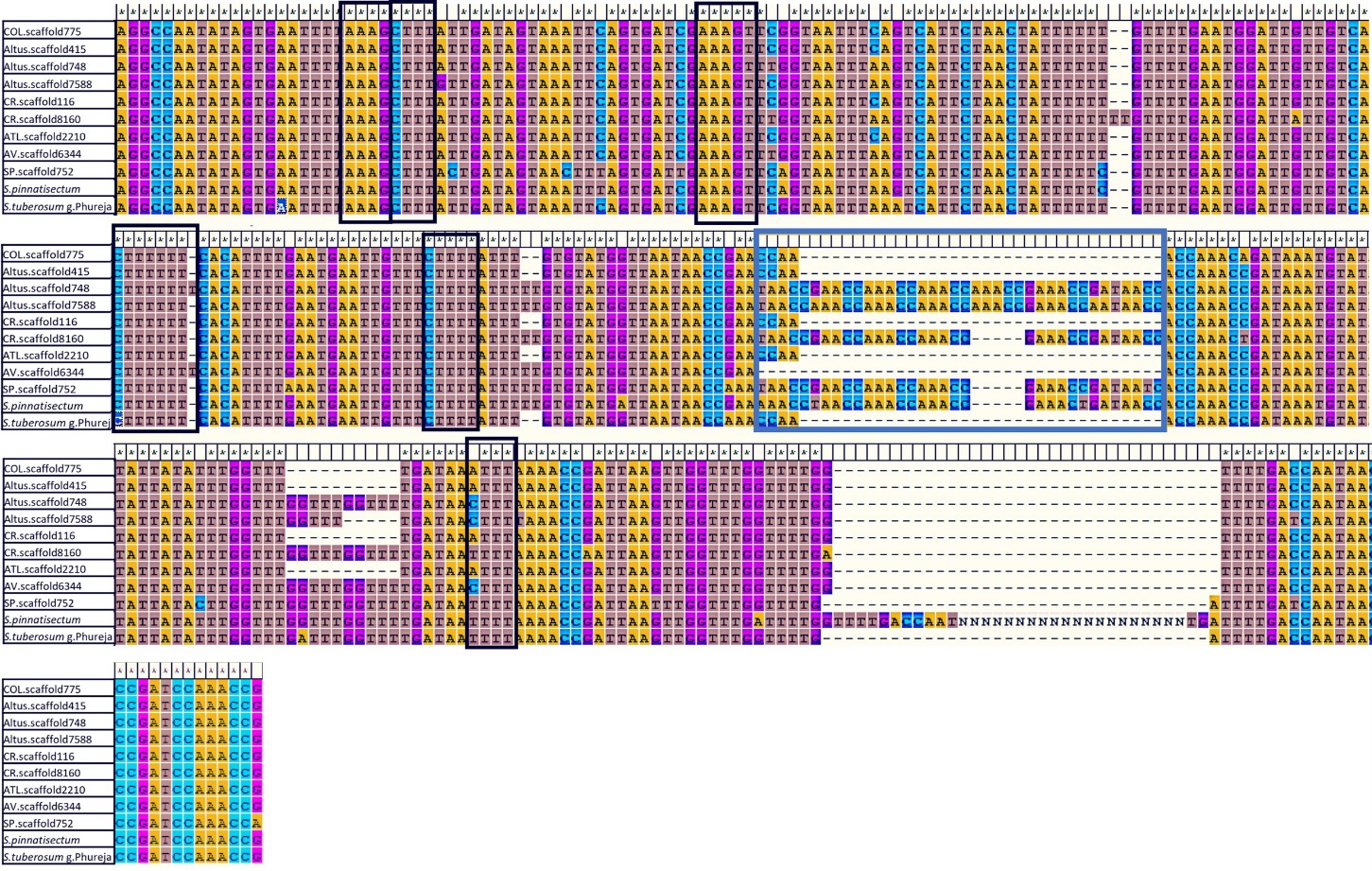


**Fig. S8.** **Allelic variation of the potato *NITRATE REDUCTASE* promoter.**

Alignment of the putative *StNR* promoter region were StCDF1 binds according to the DAP-Seq results. Sequences were extracted from the potato cultivars *Colomba*, *Altus*, *Castle Russet*, *Atlantic*, *Avenger*, and *Spunta*, the wild potato *S. pinnatisectum* and the *S. tuberosum* group Phureja DM1-3 reference genome, and they were aligned using MegAlign Pro 13 software. Col: Colomba, CR: Castle Russet, ATL: Atlantic, AV: Avenger and SP: Spunta. Black squares represent the StCDF1 binding sites (AAAGT), and the blue square the insertion found in some cultivars and *S. pinnatisectum.*


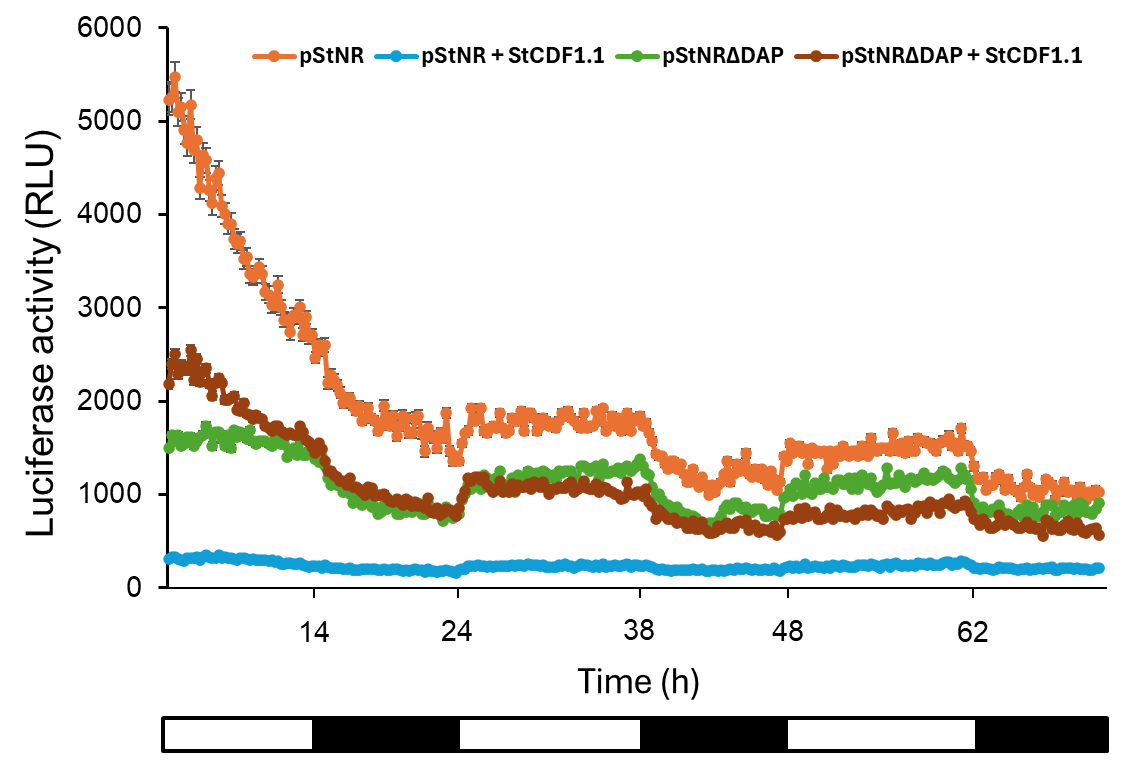


**B**

**A**

**Fig. S9.** **Removal of StCDF1-binding elements reverses the StCDF1.1 repression of NR/NIA1**

**(A)** A transient activity assay using the StNR promoter with eliminated StCDF1 binding elements (pStNR^ΔDAP^) from the DAPseq experiment shows no repression by StCDF1 compared to the intact promoter, and it remains positively regulated by StNLP7. **(B)** The pStNR^ΔDAP^ promoter also exhibits a diurnal expression pattern, similar to the intact pStNR, during both light and dark periods.


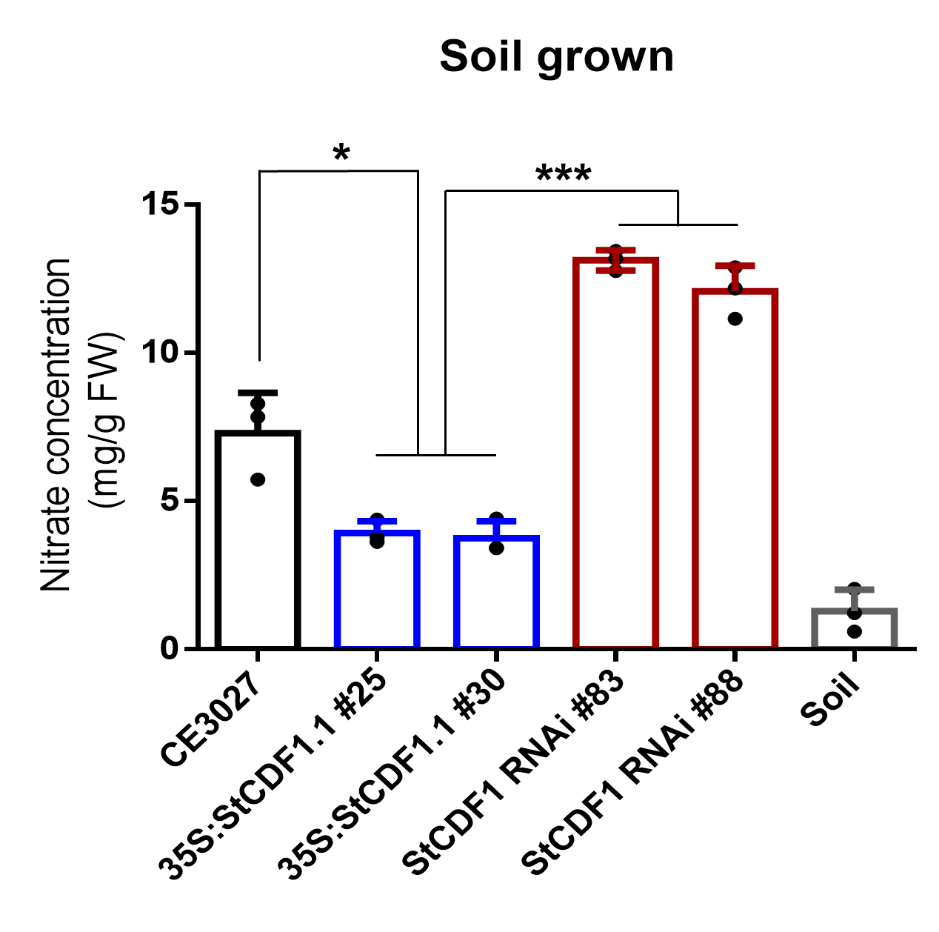


**Fig. S10****.** **Nitrate quantification (mg/g FW) of soil grown potato plants.** CE3027 (control), *35S:StCDF1.1* (line #25 and #30), *StCDF1* RNAi (line #83 and #88) plants and soil mixture. Data represent the mean ± SD of n=3 plants. Asterisks indicate significant differences for each genotype (p<0.05), *t*-test.


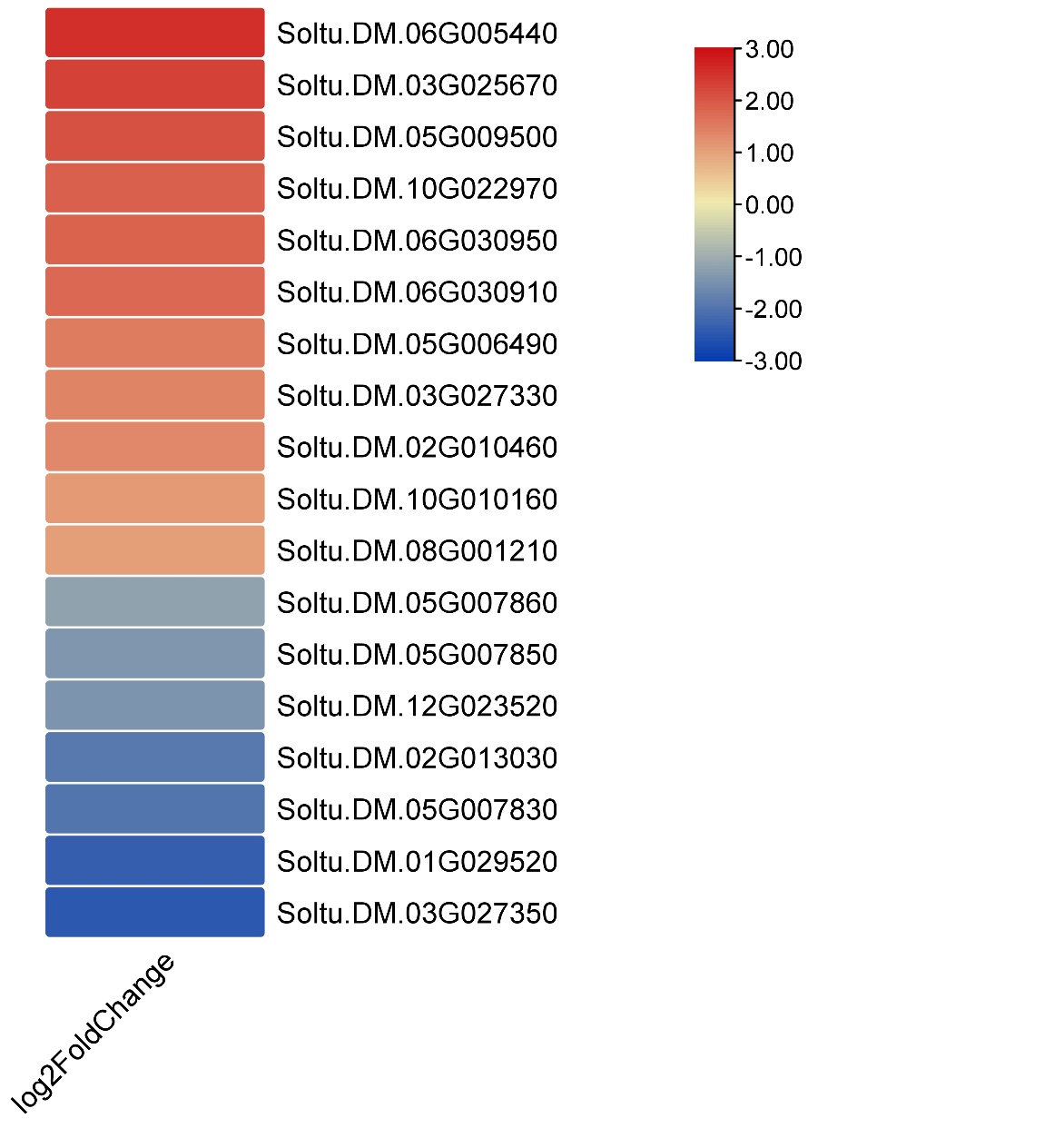


**Fig. S11.** **Potato** **nitrate transporters differentially expressed in *StCDF1ox* vs *StCDF1rnai* RNAseq dataset**.


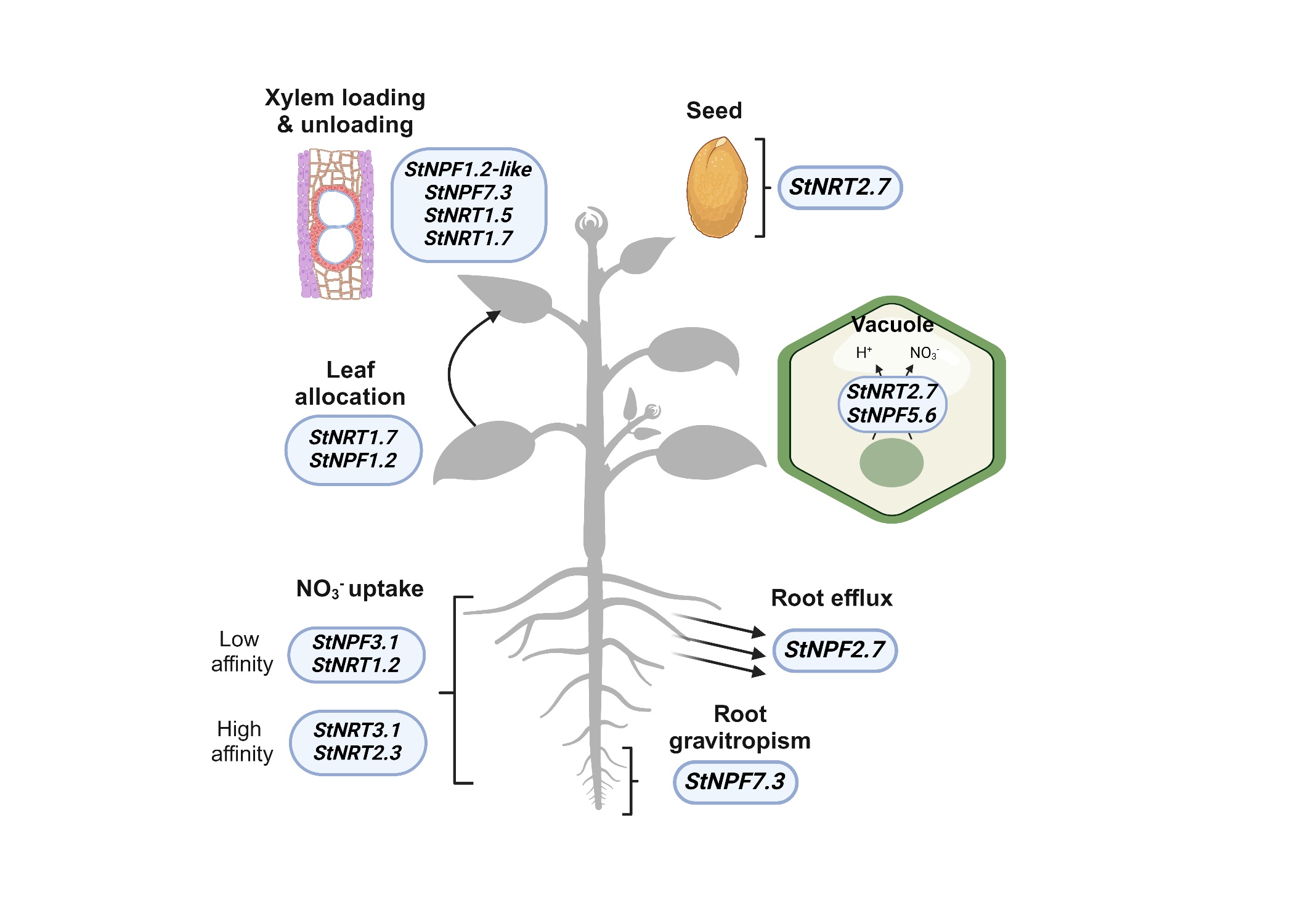


**Fig. S12.** **Physiological function of Nitrate transporters**. In NO_3_^-^ uptake, Xylem loading and unloading, Root-to-shoot transport, Nitrate efflux from roots, Leaf allocation and Root gravitropism.
